# Enhanced Osprey Optimization Algorithm for Global Optimization with Application to PEM Fuel Cell Parameter Identification

**DOI:** 10.3390/biomimetics11080545

**Published:** 2026-08-03

**Authors:** Yacine Bouali, Basem Alamri

**Affiliations:** 1 Laboratory of Research in Electrical Systems and Energy Conversion (LRSECE), Department of Electrical Engineering, National Higher School of Advanced Technologies (ENSTA), P.O. Box 474, Algiers 16001, Algeria; 2 Department of Electrical Engineering, College of Engineering, Taif University, P.O. Box 11099, Taif 21944, Saudi Arabia; b.alamri@tu.edu.sa

**Keywords:** osprey optimization algorithm (OOA), enhanced osprey optimization algorithm (EOOA), CEC2022 benchmark functions, proton exchange membrane fuel cell (PEMFC), global optimization, parameter identification

## Abstract

Bio-inspired metaheuristic algorithms, which emulate natural predatory and evolutionary behaviors, play a crucial role in solving complex engineering problems, such as the accurate parameter extraction of proton exchange membrane fuel cells (PEMFCs). However, many existing optimization algorithms suffer from premature convergence, premature stagnation in local minima, and limited accuracy. Among these algorithms, the Osprey Optimization Algorithm (OOA) has shown promising performance. In this paper, an Enhanced Osprey Optimization Algorithm (EOOA), an improved variant of the conventional OOA, is proposed. The performance of the proposed algorithm is first evaluated using the CEC2022 benchmark functions. Subsequently, the EOOA is applied to the problem of PEMFC parameter extraction for two commercial stacks, namely NedStack PS6 and Ballard Mark V. The results demonstrate that the EOOA outperforms the original OOA and four other metaheuristic algorithms, ranking first in 11 out of 12 CEC2022 benchmark functions. Furthermore, the EOOA shows superior performance in PEMFC parameter identification compared to the OOA and other methods reported in the literature. Specifically, the proposed algorithm achieves a sum of squared errors (SSE) of 2.065 for the NedStack PS6 and 0.81 for the Ballard Mark V. These results indicate that the EOOA has strong potential for application to other optimization problems beyond PEMFC parameter extraction.

## 1. Introduction

The growing demand for energy across multiple sectors, together with the rapid depletion of fossil fuel resources and the impacts of climate change on the environment, has been widely recognized as a key driver of the energy transition over the past decade [1]. In this context, the development of sustainable and renewable energy technologies has become a major research priority. Among the available options, Proton Exchange Membrane Fuel Cells (PEMFCs) have attracted considerable attention for renewable energy systems, fuel cell vehicles (FCVs) and transportation applications due to their high energy conversion efficiency, relatively low operating temperature, rapid start-up characteristics, and minimal pollutant emissions [2,3,4,5].

Electricity in PEMFC systems is generated through an electrochemical process in which hydrogen and oxygen react across the membrane, resulting in the direct conversion of chemical energy into electrical energy. The reaction products consist primarily of water, accompanied by the release of heat [6]. However, the performance and efficiency of PEMFCs strongly depend on several operating parameters and design variables, making their optimization a complex task. Consequently, advanced optimization techniques, particularly metaheuristic algorithms, have attracted considerable attention for enhancing PEMFC performance and identifying optimal operating conditions.

For the accurate design and analysis of PEMFC systems, it is essential to develop reliable mathematical models and perform detailed simulations [7]. However, the highly nonlinear behavior and operating-condition-dependent dynamics of PEMFCs make it challenging to obtain simulation results that closely match the actual performance of the system [8]. Consequently, parameter extraction has emerged as an important research topic in the field [9]. In this context, the parameter identification problem is commonly formulated as an optimization problem, where the objective is to determine a set of model parameters that minimize the discrepancy between the model outputs and the experimental data [10]. Due to the complexity and nonconvex nature of this problem, metaheuristic optimization algorithms have been widely adopted as effective tools for solving the parameter estimation problem in PEMFC models.

Biomimetics, which translates strategies observed in biological systems into engineering solutions, has become a powerful paradigm for solving complex optimization problems. Nature-inspired metaheuristic algorithms emulate biological behaviors to efficiently explore nonlinear and multimodal search spaces without requiring derivative information, making them particularly well suited for engineering applications such as PEMFC parameter identification [11]. Their effectiveness relies on balancing exploration of the search space and exploitation of promising solutions, which helps avoid premature convergence and increases the likelihood of finding global optima [12]. Many modern metaheuristics also incorporate adaptive mechanisms that enhance robustness in dynamic environments [13]. As a result, they have been successfully applied in various fields such as engineering design [14], machine learning [15], scheduling [16], data mining [17], and parameter identification [18,19].

Recent studies have extensively explored advanced optimization and hybrid intelligent techniques for accurate parameter estimation of PEMFC models. These include the enhanced artificial bee colony optimization [20], tianji’s horse racing optimization [6], mirage search optimizer [21], the cloud drift optimization (CDO) algorithm [22], parrot optimizer [23], circle search algorithm (CSA) [24], the artificial rabbit optimization algorithm [25], the electric eel foraging optimization algorithm [26], and the archimedes optimization algorithm [27].

In [28], a differential evolution (DE) algorithm with a dynamic crossover strategy was proposed to enhance both exploration and exploitation capabilities during the optimization process, leading to highly accurate parameter extraction for commercial PEMFC stacks such as the NedStack PS6 and BCS 500W. The algorithm integrates adaptive mutation strategies and a dynamic crossover mechanism, significantly improving convergence stability and reducing the root mean square error compared with several state-of-the-art optimization techniques. Similarly, metaheuristic-based approaches such as the dimension learning-based modified grey wolf optimizer (DLHMGWO) [29] and adaptive particle swarm optimization (APSO) [30] have been employed to address the nonlinear and complex characteristics of PEMFC models. These methods enhance population diversity, balance exploration and exploitation, and improve convergence behavior, enabling accurate estimation of key model parameters even in the presence of nonlinearities and noisy data. Other studies have introduced novel optimization frameworks to further improve parameter identification performance. For instance, the quantum-encoded pathfinder algorithm (QPFA) proposed in [31] incorporates concepts from quantum computing to increase population diversity and avoid premature convergence, resulting in improved stability and precision in extracting PEMFC parameters. In addition, hybrid data-driven strategies combining machine learning and metaheuristic optimization have gained attention. The feedforward neural network-pelican optimization algorithm (FNN-POA) proposed in [32] employs neural networks for data preprocessing and noise reduction before performing parameter identification, significantly enhancing robustness and optimization quality. Likewise, the CNN-assisted improved pufferfish optimization algorithm (CNN–IPOA) introduced in [33] integrates convolutional neural networks with an enhanced optimization mechanism to improve the predictive capability of PEMFC models and achieve voltage-current characteristics closely matching experimental data. More recently, hybrid optimization schemes such as the aquila optimizer-arithmetic algorithm optimization (AOAAO) [34] have been developed to further improve parameter estimation performance. By combining the strengths of two optimization algorithms and introducing mutation strategies, AOAAO achieves superior convergence speed, high accuracy, and improved computational efficiency, demonstrating its effectiveness for reliable PEMFC modeling across multiple operating conditions.

In this paper, a new proposed algorithm is investigated. The proposed method is an enhancement of the osprey optimization algorithm (OOA) [35], referred to as the enhanced osprey optimization algorithm (EOOA), in which an additional phase is introduced to the conventional OOA, the additional phase is based completly on the differential evolution algorithms to address the limitations of the original OOA. The proposed algorithm is first evaluated using the CEC2022 benchmark functions, and then applied to extract the parameters of two commercial PEMFC stacks.

The rest of this paper is organized as follows. Section 2 reviews the existing literature on modifications and improvements of the OOA. Section 3 presents the proposed EOOA, including an overview of the original OOA and the proposed enhancement strategies. Section 4 evaluates the performance of the proposed algorithm using the CEC2022 benchmark functions. Section 5 formulates the PEMFC parameter identification problem, including the PEMFC mathematical model and the objective function formulation. Section 6 presents and discusses the results obtained for the PEMFC parameter identification problem using the proposed approach. Finally, Section 7 concludes the paper and summarizes the main findings.

## 2. Review of Osprey Optimization Algorithm Modifications

Several studies have proposed enhancements to the OOA to address its core limitations: slow convergence, susceptibility to local optima, and weak exploitation. Common improvement strategies include incorporating Lévy flight [36,37] to boost exploration, attack-defense strategies [38] for better convergence, Tent mapping and Gaussian distribution [39] for improved initialization and perturbation, and hybrid combinations with other algorithms such as the parrot optimizer [40] and kookaburra optimization algorithm [41]. These modifications collectively yield faster convergence, greater solution accuracy, and improved robustness across diverse problem types.

The improved OOA variants have been applied to a wide range of engineering domains. In energy systems, they are used for PEMFC electrochemical parameter identification [38], optimal reactive power dispatch with electric vehicle integration [42], and supercapacitor parameter identification [40]. In cloud and distributed computing, enhanced versions tackle task scheduling [36] and adaptive load balancing [41]. Additional applications include leukemia image classification using optimized CapsNet [43], industrial availability optimization of a stainless steel manufacturing unit [37], and mobile robot path planning [39].

In [44], the authors proposed an improved version of the OOA to address slow convergence and local optima issues. The enhancement combines three strategies: Sobol sequence initialization to increase population diversity, a Weibull distribution-based step factor to balance exploration and exploitation during position updates, and a disturbance mechanism inspired by the firefly algorithm to help escape local optima. The proposed algorithm was validated on CEC2017 benchmark functions and applied to the reactive power optimization problem in IEEE-33 and IEEE-69 distribution networks, achieving significant reductions in active power network losses.

In [45], the authors proposed a modified version of the OOA called modified OOA (MOOA) to enhance its exploration capability and avoid premature convergence. The improvement integrates three strategies: a Lévy flight mechanism to expand the search range, a Brownian motion strategy to increase population diversity, and  the roulette fitness-distance balance-based (RFDB) selection method to guide the population toward high-quality solutions and improve global search. The proposed algorithm was validated on CEC2017 and CEC2022 benchmark functions and further applied to several engineering design optimization problems, demonstrating improved convergence accuracy and stability compared with several existing metaheuristic algorithms.

In [46], the authors proposed a multi-strategy improved version of the OOA to enhance global search ability and convergence accuracy. The modification integrates Circle chaotic mapping for population initialization to increase diversity, a dynamically adjustable elite guidance mechanism to guide the search toward promising solutions during later iterations, and a dynamic chaotic weight factor to strengthen local search capability. The proposed algorithm was validated on benchmark functions and further applied to optimize hyperparameters of an long short-term memory (LSTM) model for power load forecasting, as well as several engineering design optimization problems.

In [47], the authors proposed an improved version of the OOA based on a two-color complementary mechanism to enhance optimization accuracy and avoid local optima. The method first employs logistic chaotic mapping combined with the good point set method for population initialization and divides the population into four color groups to increase diversity. Different strategies are then assigned to each group, including the core OOA exploration, mechanisms inspired by the harris hawks optimization algorithm, roaming search strategies, optimized spiral search, and perturbation from the firefly algorithm to strengthen exploration and local search capabilities. The proposed algorithm was validated on CEC2020 and CEC2022 benchmark functions and applied to several engineering optimization design problems.

In [48], the authors proposed a multi-strategy improved version of the OOA to address the imbalance between exploration and exploitation and reduce premature convergence. The improvement incorporates Fuch chaotic mapping for population initialization to enhance diversity, an adaptive weighting factor to improve convergence accuracy during exploration, and a Cauchy mutation strategy to avoid local optima during exploitation. Additionally, the warner mechanism from the sparrow search algorithm is integrated to balance global and local search. The proposed method was evaluated on benchmark and CEC2017 test functions and applied to the three-bar truss engineering design optimization problem.

In [49], the authors proposed a multi-strategy improved version of the OOA to enhance population diversity and prevent premature convergence. The improvement includes Piecewise chaotic mapping for population initialization, an adaptive Lévy-based exploration strategy guided by a leader mechanism, and a trigonometric random disturbance strategy to increase diversity. Moreover, nonlinear control parameters and an elite leader-based position update mechanism are introduced to balance exploration and exploitation and accelerate convergence. The improved OOA was tested on benchmark functions and applied to the UAV path planning optimization problem.

In [50], the authors proposed an enhanced hybrid multi-objective version of OOA (EHMOOOA) to improve optimization performance in complex multi-objective problems. The method integrates quasi-oppositional-based learning for improved population diversity, a hybrid mutation mechanism based on the differential evolution algorithm, and a Brownian motion strategy to enhance exploitation during the final search stage. The proposed algorithm was applied to the optimal sizing of an off-grid hybrid renewable energy system consisting of photovoltaic panels, wind turbines, and battery storage, aiming to minimize loss of power supply probability, levelized cost of energy, and net present cost.

Across all studies, the modified OOA variants consistently outperform both the original OOA and competing metaheuristics. The results confirm that targeted structural enhancements; whether through chaotic maps, hybrid architectures, or adaptive strategies, significantly extend the OOA’s applicability and effectiveness in solving complex real-world optimization problems. Table 1 summarizes the main modifications proposed in the reviewed studies, along with their corresponding applications. However, despite these improvements, many modified OOA variants still face challenges in maintaining a proper balance between exploration and exploitation and may suffer from premature convergence in complex optimization problems. Therefore, this paper proposes an enhanced osprey optimization algorithm (EOOA) to further improve convergence performance and solution quality.

## 3. Enhanced Osprey Optimization Algorithm (EOOA)

### 3.1. Overview of the Original Osprey Optimization Algorithm

The hunting and fish-carrying behavior of the osprey represents an intelligent natural strategy that inspires the OOA [35]. By mathematically modeling these behaviors, this optimization method was developed. In this section the modeling process used to design the original OOA is described.

In the original OOA, the search process starts with the random initialization of *N* agents distributed within the feasible search space. Each agent represents a potential solution to the optimization problem. The initial position of the *i*-th agent in the *d*-th dimension is generated as

(1) $$x_{i,d}=lb_{d}+\mathrm{rand}\;\left(ub_{d}-lb_{d}\right),\;i=1,2,\ldots ,N,\;d=1,2,\ldots ,D$$

where *D* denotes the dimensionality of the problem (number of decision variables). The term $x_{i,d}$ indicates the coordinate of agent *i* along dimension *d*, while $ub_{d}$ and $lb_{d}$ represent the upper and lower bounds of that dimension, respectively. The function rand generates a random number uniformly distributed in the interval (0,1).

Each agent $x_{i}$ is therefore a vector composed of *D* elements, and the entire population of agents can be arranged into a matrix of size N×D as follows:

(2) $$x=\left[\begin{array}{cccc} x_{1,1} & x_{1,2} & \ldots & x_{1,D}\\ x_{2,1} & x_{2,2} & \ldots & x_{2,D}\\ \vdots & \vdots & \ddots & \vdots \\ x_{N,1} & x_{N,2} & \ldots & x_{N,D} \end{array}\right].$$

Once the initial population is generated, the fitness values of all agents are computed using the objective function and stored in the following vector:

(3) $$F={\left[\begin{array}{c} F(x_{1})\\ F(x_{2})\\ \vdots \\ F(x_{N}) \end{array}\right]}_{N\times 1}$$

where *F* is a vector of size N×1 used to store and update the fitness values of all agents in the population.

**Phase 1:** In this phase, a global search is conducted to prevent the algorithm from becoming trapped in local optima. Ospreys simulate the hunting behavior of attacking fish, enabling an extensive exploration of the search space. Initially, the positions of the fish are determined using the following equation:

(4) $$FP_{i}=\left\{x_{k}|k\in \left\{1,2,\ldots ,N\right\}\cap F_{k}<F_{i}\right\}\cup \left\{x_{best}\right\}$$

where $FP_{i}$ represents the selected position of the fish for the *i*-th osprey, $F_{i}$ denotes the fitness value of the *i*-th agent, and $x_{\mathrm{best}}$ corresponds to the position of the best agent in the population.

After identifying the target positions, the osprey attempts to attack them. This behavior is mathematically modeled by the following equation:

(5) $$x_{i,j}^{P1}=x_{i,j}+r_{1}\left(SF_{i,j}-I_{i,j}\times x_{i,j}\right),\;i=1,2,\ldots ,N,\;j=1,2,\ldots ,D$$

where $r_{1}$ is a random number in the interval (0,1), $SF_{i,j}$ denotes the position of the selected fish among the identified fish, and $I_{i,j}$ is a random integer in the set {1,2}. The newly generated position replaces the previous one if its fitness value is better than that of the current position, as defined in the following equation:

(6) $$x_{i}=\left\{\begin{array}{cc} x_{i}^{P1}, & \mathrm{if}\;F_{i}^{P1}<F_{i}\\ x_{i}, & \mathrm{otherwise} \end{array}\right.$$

where $F_{i}^{P1}$ denotes the fitness value of the newly generated position.

**Phase 2:** In this phase, the ospreys search for a suitable position after successfully hunting a fish. This stage enables the OOA to perform a local search and facilitates convergence toward the optimal solution. The corresponding mathematical model is expressed in the following equation:

(7) $$x_{i,j}^{P2}=x_{i,j}+\frac{lb_{j}+r_{2}\left(ub_{j}-lb_{j}\right)}{t},\;t=1,2,\ldots ,T_{max}$$

where $r_{2}$ is a random number in the interval (0,1), and *t* and $T_{\max}$ denote the current iteration and the maximum number of iterations, respectively. Similar to the previous phase, the newly generated individual replaces the current one if it provides a better solution, as determined by the following equation:

(8) $$x_{i}=\left\{\begin{array}{cc} x_{i}^{P2}, & \mathrm{if}\;F_{i}^{P2}<F_{i}\\ x_{i}, & \mathrm{otherwise} \end{array}\right.$$

where $F_{i}^{P2}$ denotes the fitness value of the newly generated position.

### 3.2. Proposed Enhancements to the Osprey Optimization Algorithm

The OOA [35] employs a two-phase search mechanism inspired by the hunting behaviour of ospreys, as described in the previous section. Although OOA demonstrates competitive performance on several benchmark optimization problems, it presents some structural limitations. In particular, the algorithm relies on a fixed exploration–exploitation mechanism that does not adapt dynamically during the optimization process, and it lacks explicit cross-agent information sharing among population members.

To address these limitations and improve the balance between exploration and exploitation, an additional search phase based on DE is incorporated into the algorithm. This adaptive differential evolution phase enhances population diversity and mitigates premature convergence by generating new candidate solutions through mutation and crossover operations among multiple agents.

#### 3.2.1. Adaptive Control Parameter

A nonlinear adaptive factor is introduced to control the mutation strength during the optimization process. The parameter is defined as

(9) $$\alpha_{t}=\exp\left(-\lambda {\left(\frac{t}{T_{\max}}\right)}^{2}\right)$$

here, λ is a decay coefficient controlling the steepness of the curve, ranging from 2 to 4.

Using this factor, the DE scaling factor is dynamically adjusted as

(10) $$F_{de}\left(t\right)=F_{\min}+\left(F_{\max}-F_{\min}\right)\alpha_{t}$$

where $F_{\min}$ and $F_{\max}$ represent the minimum and maximum mutation scaling factors, respectively; with $F_{\min}=0.3$ and $F_{\max}=0.9$ in this paper. This adaptive strategy enables large mutation steps during early iterations (exploration) and smaller perturbations in later iterations (exploitation).

#### 3.2.2. Mutation Operator

Three individuals $x_{r1}$, $x_{r2}$, and $x_{r3}$ are randomly selected from the population such that $r_{1}\neq r_{2}\neq r_{3}\neq i$. A mutant vector is then generated as follows:

(11) $$v_{i}=x_{r1}+F_{de}\left(t\right)\left(x_{r2}-x_{r3}\right)$$

where $v_{i}$ represents the mutant vector and $F_{de}\left(t\right)$ is the adaptive scaling factor defined previously.

#### 3.2.3. Crossover Operator

To increase diversity, a binomial crossover operator is applied between the mutant vector and the current solution $x_{i}$. The trial vector $u_{i}$ is generated as

(12) $$u_{i,j}=\left\{\begin{array}{cc} v_{i,j}, & \mathrm{if}\;rand_{j}\leq CR\\ x_{i,j}, & \mathrm{otherwise} \end{array}\right.$$

here, CR denotes the crossover probability, which is ranged from 0.1 to 0.9, and $rand_{j}$ is a uniformly distributed random number in the interval (0,1).

#### 3.2.4. Selection Mechanism

A greedy selection strategy is used to determine whether the generated trial vector replaces the current solution. The update rule is defined as

(13) $$x_{i}=\left\{\begin{array}{cc} u_{i}, & \mathrm{if}\;F\left(u_{i}\right)<F\left(x_{i}\right)\\ x_{i}, & \mathrm{otherwise} \end{array}\right.$$

where F(·) represents the objective function value.

The introduction of this adaptive DE phase improves the algorithm’s global search capability during early iterations while allowing fine local adjustments in later stages, thus enhancing the overall convergence performance.

**Figure 1:**
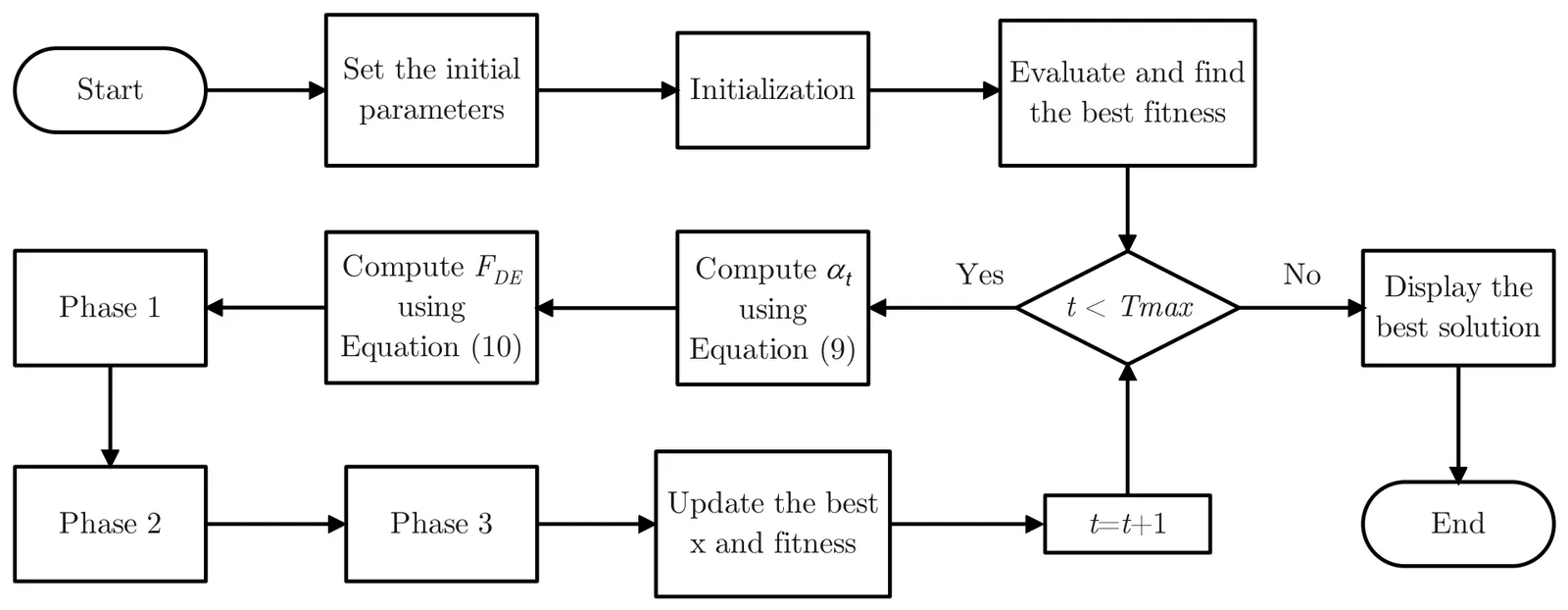
The flowchart of EOOA.

The pseudocode and the flowchart of the EOOA are presented in Algorithm 1 and Figure 1, respectively.
**Algorithm 1** Enhanced Osprey Optimization Algorithm (EOOA) pseudo-code  1:**Input:** *N*, $T_{\max}$, lb, ub, F(·), and *D*  2:**Output:** best solution $x^{*}$, best fitness $F^{*}$  3:Initialize population $x_{i}∼U\left[lb,ub\right]$, evaluate $F(x_{i})$, i=1,…,N  4:$x^{*}\leftarrow \arg\min_{i}f\left(x_{i}\right)$,    $f^{*}\leftarrow f\left(x^{*}\right)$  5:**for** t=1 **to** $T_{\max}$ **do**  6:      Compute $\alpha_{t}$ as in Equation (9)                                                       ▹ *Adaptive parameter*  7:      Compute $F_{\mathrm{DE}}$ as in Equation (10)                                                      ▹ *DE scaling factor*  8:      **for** i=1 **to** *N* **do**  9:             **Phase 1: Exploration**10:             Determine the fish position using Equation (4).11:             Compute the new osprey position using Equation (5).12:             Update the *i*-th osprey using Equation (6).13:             **Phase 2: Exploitation**14:             Compute the new osprey position using Equation (7).15:             Update the *i*-th osprey using Equation (8).16:             **Phase 3: DE Mutation & Crossover**17:             Randomly select distinct $r_{1},r_{2},r_{3}\in \left\{1,\ldots ,N\right\},\;r_{1},r_{2},r_{3}\neq i$18:             Calculate the mutant vector using Equation (11)                                 ▹ *Mutation*19:             Calculate the trial vector $u_{i,j}$ using Equation (12)                                 ▹ *Crossover*20:             Update $x_{i}$ using Equation (13)                                                      ▹ *Greedy selection*21:      **end for**22:      Update $x^{*}$, $F^{*}$ from current population23:**end for**24:**return** $x^{*}$, $F^{*}$

The MATLAB and Python 3.8+ implementations of the proposed EOOA are publicly available at: https://github.com/Yacine-Bouali/Enhanced-Osprey-Optimization-Algorithm-EOOA--Matlab-code (accessed on 15 June 2026).

### 3.3. Time Complexity Analysis

Time complexity is a critical metric for evaluating the computational efficiency and convergence speed of an optimization algorithm. For the original OOA, the population initialization phase requires O(N×D) operations, where *N* denotes the number of search agents and *D* denotes the problem dimensionality. Since each of the two position-update phases incurs a cost of O(N×D) per iteration over $T_{\max}$ iterations, the overall time complexity of the original OOA is:

$$O\;\left(N\times D\times (1+2T_{\max})\right)$$

In the proposed EOOA, a third phase is introduced. As demonstrated in the preceding analysis, Phase 3 shares the same per-iteration asymptotic cost, O(N×D), as Phases 1 and 2, since its dominant operation remains a single objective function evaluation per agent. Consequently, the total time complexity of EOOA is:

$$O\;\left(N\times D\times (1+3T_{\max})\right)$$

## 4. Results of EOOA

### 4.1. Performance Evaluation on the CEC2022 Benchmark Functions

To evaluate the performance of the proposed EOOA, it was compared with several well-known optimization algorithms, including OOA, genetic algorithm (GA) [51], sine cosine algorithm (SCA) [52], artificial bee colony (ABC) algorithm [53,54], and harris hawks optimization (HHO) [55]. The experiments were conducted using the CEC2022 benchmark test functions, listed in Table 2. The corresponding control parameters of these algorithms are presented in Table 3.

All experiments were carried out on a personal computer running a 64-bit Windows 10 operating system, equipped with an Intel Core i5-3230M CPU operating at 2.60 GHz and 8 GB of RAM. To enable a controlled comparison, each algorithm was executed for 30 independent runs using the same population size of 50 and a maximum number of 500 iterations. However, matching the population size and the number of iterations alone does not necessarily ensure an identical computational budget, as different algorithms may perform different numbers of objective function evaluations per iteration. This iteration-matched comparison framework was retained to maintain consistency with the experimental settings commonly adopted in the metaheuristics literature.

Table 4 summarizes the statistical performance of the proposed EOOA algorithm and five optimization methods (OOA, GA, SCA, ABC, and HHO) on the CEC2022 benchmark functions. For each test function, the best, worst, mean, and standard deviation (Std) values obtained over 30 independent runs are reported. The Friedman ranking test is also included to provide a comparative statistical evaluation of the algorithms. The best mean value for each function is highlighted in bold.

The results clearly demonstrate the strong optimization capability of the proposed EOOA. In particular, EOOA achieves the best mean performance on 11 out of the 12 benchmark functions ($F_{1}$–$F_{9}$, $F_{11}$, and $F_{12}$), indicating its superior ability to locate high-quality solutions across different types of optimization landscapes. Moreover, the standard deviation values obtained by EOOA are generally smaller than those of the competing algorithms, which reflects a stable and consistent search behavior over multiple independent runs.

For unimodal and relatively simple problems, such as $F_{1}$–$F_{4}$, EOOA shows excellent convergence accuracy, achieving results very close to the global optimum while maintaining low variance. This indicate that the exploitation capability of the proposed algorithm is effective in refining candidate solutions in the final stages of the search process. In contrast, algorithms such as GA and OOA exhibit significantly larger mean values and higher standard deviations, indicating weaker convergence precision.

On more complex functions, such as $F_{5}$–$F_{9}$, EOOA continues to outperform most competitors by maintaining significantly lower mean objective values. Highlights the robustness of EOOA in handling complex and multimodal search spaces where several algorithms suffer from premature convergence or unstable behavior.

Although ABC slightly outperforms EOOA on function $F_{10}$, the proposed algorithm still achieves competitive results and ranks among the top performers. This demonstrates that EOOA maintains balanced exploration and exploitation capabilities across different problem characteristics.

The superiority of EOOA is further confirmed by the Friedman statistical ranking. As shown in the last rows of Table 4, EOOA obtains the best average rank of 1.16 and is placed in the first overall position among all compared algorithms. ABC and SCA follow in the second and third positions, respectively, while OOA and GA show weaker performance. These results validate the effectiveness and robustness of the proposed EOOA for solving complex global optimization problems.

**Figure 2:**
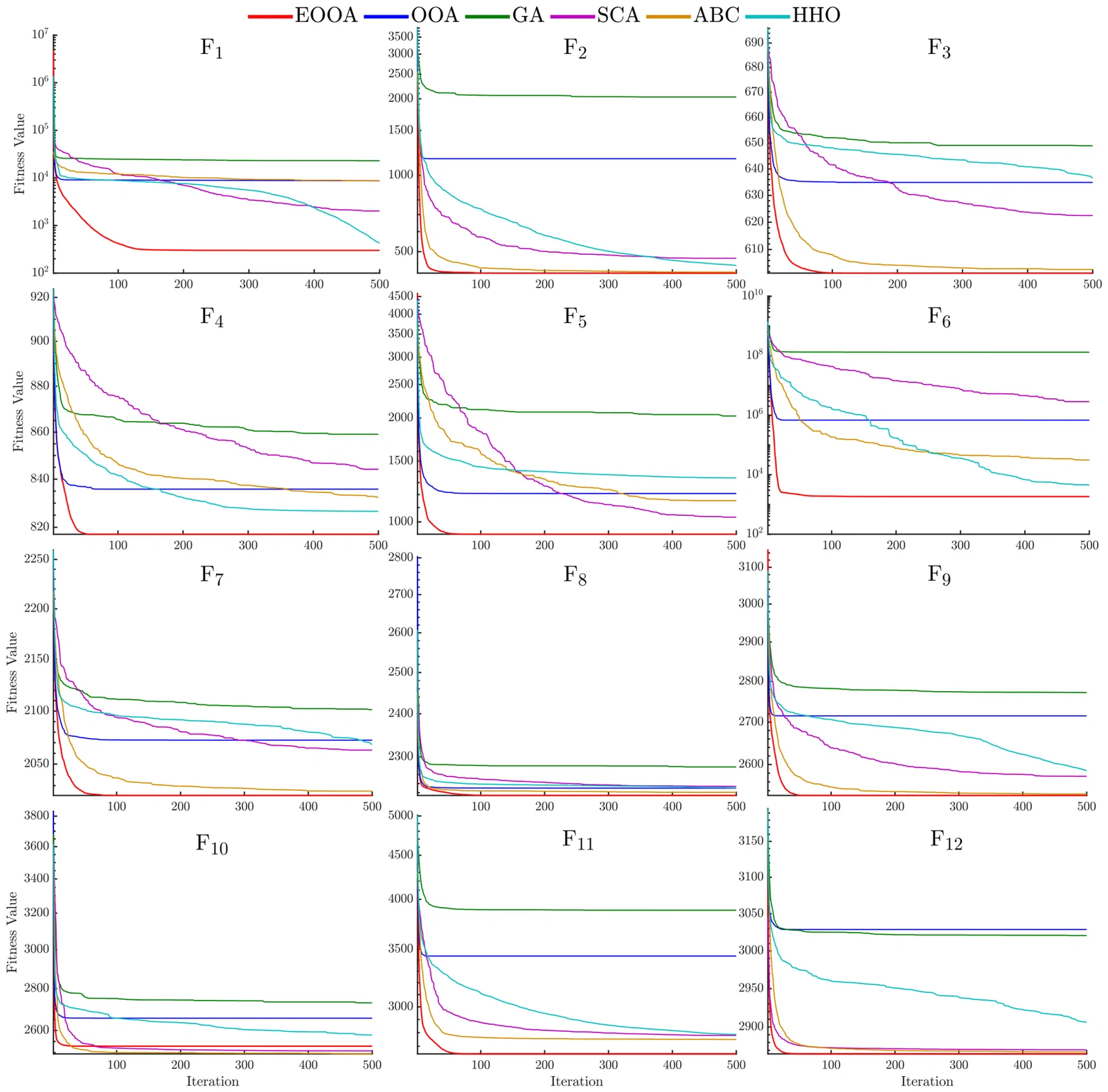
The convergence curves of EOOA and others algorithms for CEC2022.

Figure 2 illustrates the convergence behavior of EOOA and the competing algorithms on the CEC2022 benchmark functions. As observed, the proposed EOOA demonstrates a faster convergence speed and achieves lower fitness values compared with most of the other algorithms in the majority of test functions; indicating strong exploration capability. Moreover, EOOA maintains a steady improvement in later iterations, reflecting effective exploitation and good search stability. In contrast, several competing algorithms either converge more slowly or become trapped in higher fitness regions.

**Figure 3:**
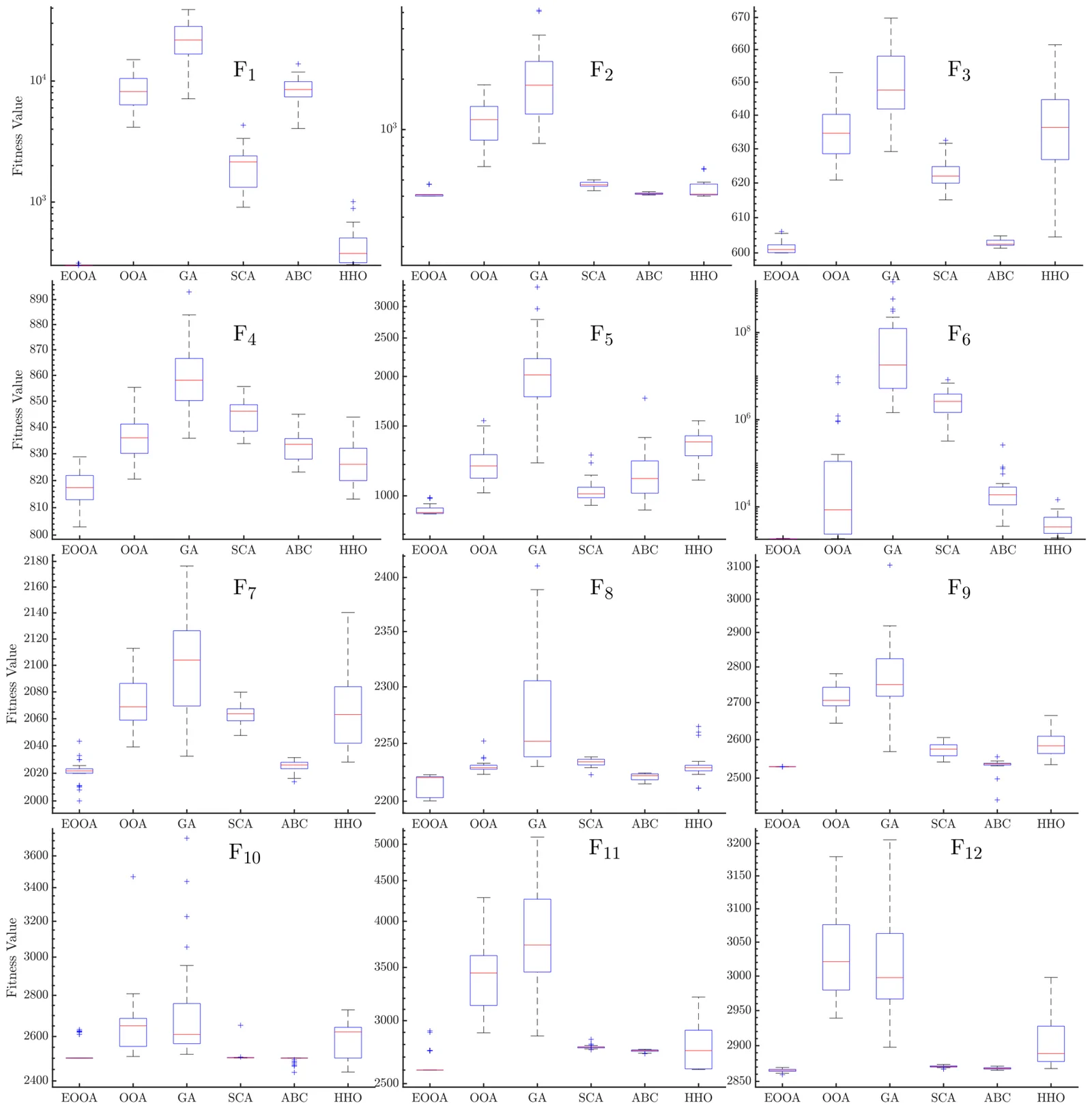
The box plot of EOOA and other algorithms for CEC2022.

Figure 3 presents the box plots of EOOA and the compared algorithms on the CEC2022 benchmark functions. As observed, EOOA generally produces lower median fitness values and smaller interquartile ranges on most functions, indicating better solution quality and higher stability. In contrast, several competing algorithms show larger variability and higher median values. These results demonstrate that EOOA provides more consistent and reliable performance across different optimization problems.

**Figure 4:**
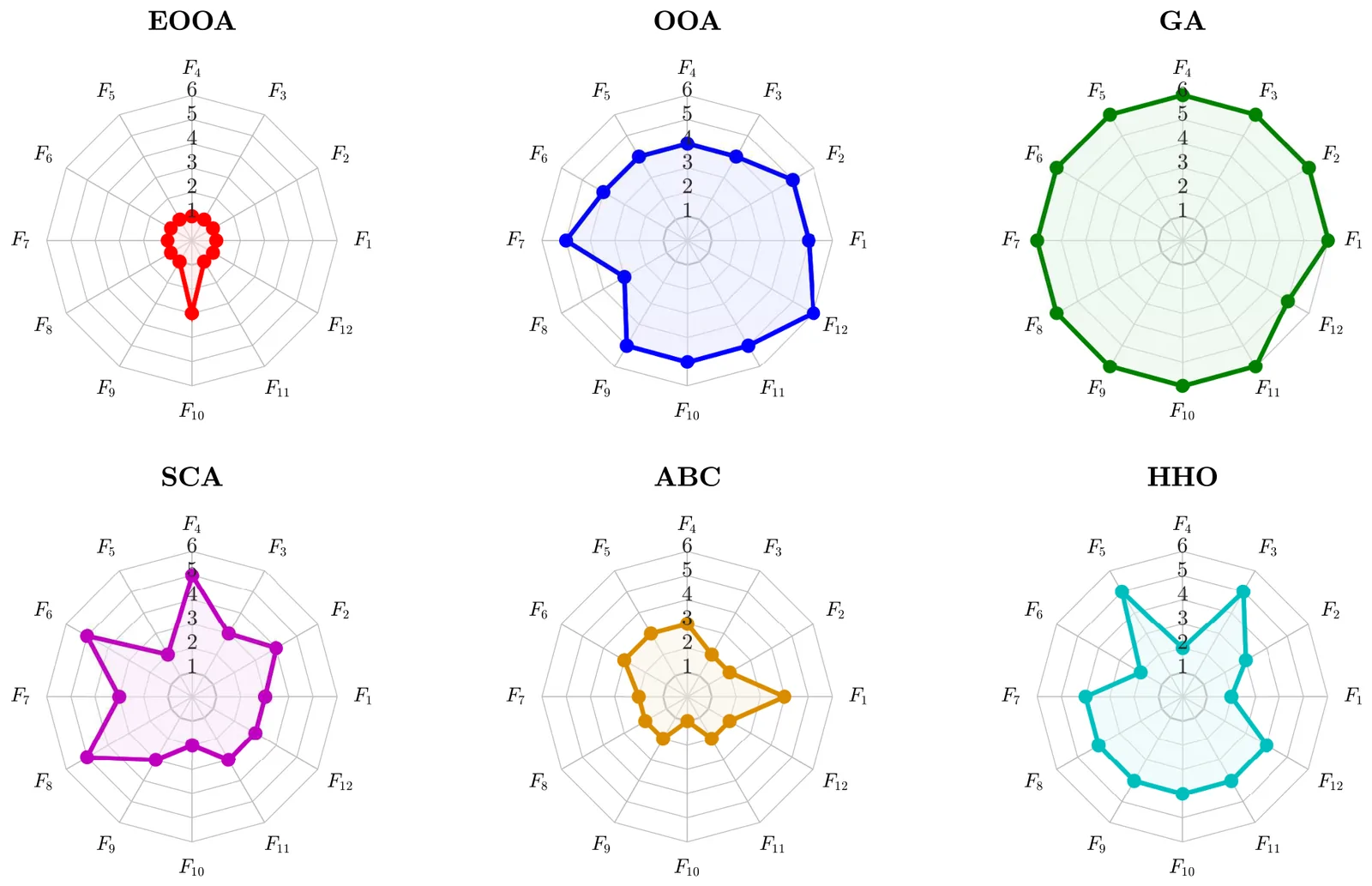
Radar plots of EOOA and others algorithms for CEC2022.

Figure 4 illustrates the radar plots representing the ranking performance of EOOA and the competing algorithms on the CEC2022 benchmark functions. As shown, EOOA consistently achieves the lowest rank values across most functions, indicating superior performance compared with the other algorithms. In contrast, GA and OOA exhibit higher rank values on several functions, reflecting weaker optimization performance. The radar plots visually confirm the dominance and robustness of EOOA over the competing methods.

To further validate the statistical significance of the obtained results, the Wilcoxon rank-sum test was performed between the proposed EOOA and the competing algorithms (OOA, GA, SCA, ABC, and HHO). The resulting *p*-values are reported in Table 5. In this table, the symbols “+”, “–”, and “=” indicate that EOOA performs significantly better than, worse than, or statistically equivalent to the compared algorithm, respectively.

As shown in Table 5, the majority of the *p*-values are extremely small (mostly lower than 0.05), indicating that the performance differences between EOOA and the other algorithms are statistically significant. In particular, EOOA significantly outperforms OOA, GA, and HHO on all benchmark functions, as indicated by the consistent “+” symbols across the table.

Similarly, when compared with SCA, the proposed EOOA achieves significantly better results on most test functions, with the exception of $F_{10}$ where SCA slightly outperforms EOOA. In the comparison with ABC, EOOA demonstrates superior performance on almost all functions, while both algorithms exhibit statistically equivalent performance on $F_{10}$.

These results confirm that the improvements obtained by the proposed EOOA are not due to random variations but rather reflect a statistically significant enhancement in optimization performance.

Table 6 reports the mean CPU execution time over 30 independent runs for all algorithms on the CEC2022 benchmark functions. As expected, EOOA requires more computation time than the original OOA due to the additional DE-based phase. Nevertheless, the execution times remain below 1 s for all test functions, indicating that the computational overhead is modest and acceptable given the observed improvements in optimization performance.

### 4.2. Parameter Sensitivity Analysis of EOOA

To evaluate the impact of internal control parameters on the performance of the EOOA algorithm, a focused sensitivity analysis was conducted. In the first test, the value of λ was varied between 2 and 4 (i.e., λ∈{2,3,4}), and for each λ value, several values of Cr (0.1, 0.5, and 0.9) were tested in order to identify the best combination of (λ, Cr). To assess generalizability across diverse problem landscapes, the analysis was applied to three representative benchmark functions $F_{4}$, $F_{6}$, and $F_{9}$.

Table 7 shows the mean and standard deviation (Std) values obtained for each function using the different combinations of (λ, Cr), along with the corresponding rank of each combination. The average rank across the three functions is also reported to identify the overall best-performing combination.

Based on these tests, the best combination that can be used in this case is λ=3 and Cr=0.9, as used in the previous subsection. It must be noted that these parameters require further tuning depending on the problem at hand and should not be treated as fixed, universally optimal values.

**Figure 5:**
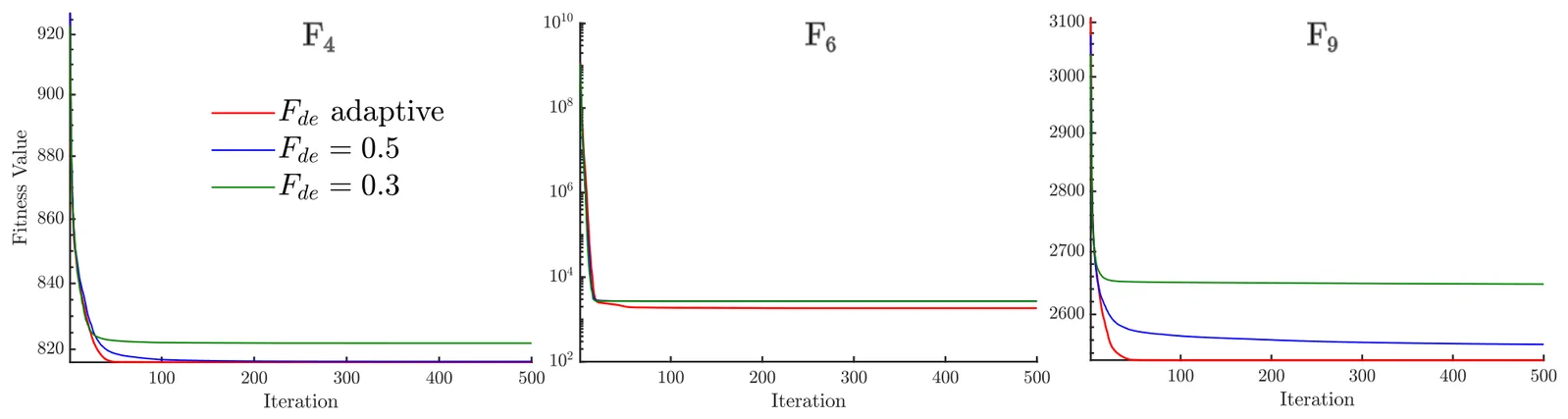
Comparison of adaptive $F_{de}$ and fixed $F_{de}$ for EOOA with respect to λ=3 and Cr=0.9 on $F_{4}$, $F_{6}$, and $F_{9}$ (CEC2022, Dim = 10).

Another parameter to test is the fixed versus adaptive form of $F_{de}$. To assess this, the results obtained in the previous test for the best combination (λ=3, Cr=0.9) with adaptive $F_{de}$ are compared against the fixed values $F_{de}=0.3$ and $F_{de}=0.5$. Figure 5 illustrates the convergence curves obtained in this test. From the figure, it is clear that the adaptive case outperforms the fixed $F_{de}$.

**Figure 6:**
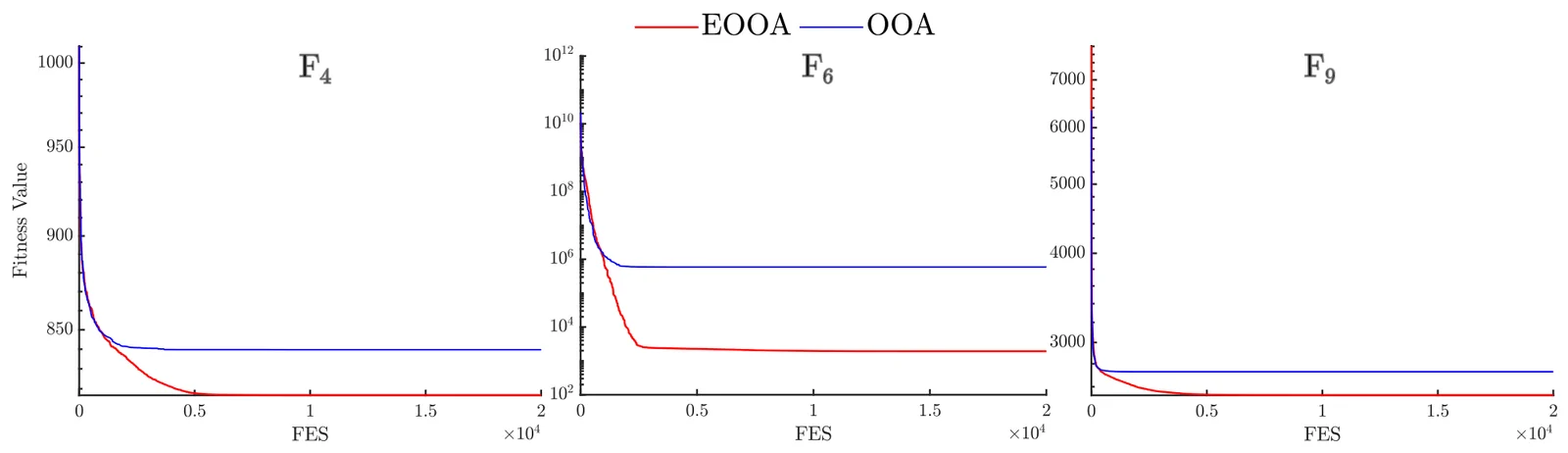
Comparison between EOOA and traditional OOA on $F_{4}$, $F_{6}$, and $F_{9}$ (CEC2022, Dim = 10), under a common budget of MaxFES = 20,000 objective function evaluations.

The evaluation of EOOA vs. OOA under the same number of objective function evaluations (FES) is presented in Figure 6, where both algorithms run on the same FES budget (MaxFES = 20,000) instead of an iteration count, with the best-so-far value recorded at each evaluation. EOOA consistently outperforms OOA in all cases.

It should be noted that the equal-FES comparison was performed only between EOOA and OOA on three representative CEC2022 functions ($F_{4}$, $F_{6}$, and $F_{9}$), selected to cover a unimodal/rotated, a hybrid, and a composition function, respectively. Although EOOA consistently outperformed OOA under the same evaluation budget, these results should not be generalized to other benchmark functions, problem dimensions, or the comparisons with GA, SCA, ABC, and HHO.

To obtain the exact number of objective function evaluations, the pseudocode can be analyzed directly, since both OOA and EOOA evaluate every agent exactly once in each search phase. For the experimental settings used in this paper (N=50, $T_{\max}=500$), OOA and EOOA require 50050 and 75050 evaluations, respectively, meaning that EOOA uses a 1.5× larger evaluation budget under the same iteration count.

Table 8 shows that, under the equal-FES setting, EOOA requires only a modest increase in CPU time compared with OOA (22–40%), reflecting the additional computational cost of its DE-based enhancement while maintaining a comparable runtime.

## 5. Formulation of the PEM Fuel Cell Parameter Identification Problem

### 5.1. PEM Fuel Cell Mathematical Model

A proton exchange membrane fuel cell operates through electrochemical reactions at both electrodes. The half-reactions at the anode and cathode, along with the overall cell reaction, are described as follows:

- Anode: H_2_ → 2H+ + 2e−
- Cathode: $\frac{1}{2}$ O_2_ + 2H+ + 2e− → H_2_O
- Overall: H_2_ + $\frac{1}{2}$ O_2_ → H_2_O

To achieve practical power output levels, $N_{cell}$ cells are assembled in series to form a stack. Referring to the equivalent electrical circuit of a single PEMFC, the total output voltage of the stack *V* is expressed as:

(14) $$V=N_{cell}\;V_{cell}$$

where the individual cell voltage $V_{cell}$ is determined by subtracting the cumulative voltage losses from the reversible open-circuit potential:

(15) $$V_{cell}=E_{Nernst}-V_{act}-V_{ohm}-V_{con}$$

The thermodynamic open-circuit voltage, $E_{Nernst}$, characterizes the maximum attainable potential under equilibrium conditions (zero current). It is derived from a temperature- and pressure-corrected form of the Nernst equation, evaluated at a reference state of $T_{0}=298.15$ K and $P_{0}=1$ bar:

(16) $$\begin{array}{cc} E_{Nernst} & =1.229-0.85\times 10^{-3}\left(T-298.15\right)\\ \; & \;+4.3085\times 10^{-5}T\ln\;\left(P_{H_{2}}\sqrt{P_{O_{2}}}\right) \end{array}$$

where *T* is the operating cell temperature (K), and $P_{H_{2}}$, $P_{O_{2}}$ are the partial pressures of hydrogen and oxygen, respectively.

The partial pressure of hydrogen at the anode is given by:

(17) $$P_{H_{2}}=0.5\;R_{HA}P_{H_{2}O}\left[{\left(\exp\;\left(\frac{1.635}{T^{1.334}}\frac{I}{A}\right)\frac{R_{HA}P_{H_{2}O}}{P_{A}}\right)}^{-1}-1\right]$$

For pure oxygen supply at the cathode, the oxygen partial pressure is obtained from:

(18) $$P_{O_{2}}=R_{HC}P_{H_{2}O}\left[{\left(\exp\;\left(\frac{4.192}{T^{1.334}}\frac{I}{A}\right)\frac{R_{HC}P_{H_{2}O}}{P_{C}}\right)}^{-1}-1\right]$$

When air is used as the oxidant instead of pure oxygen, $P_{O_{2}}$ is recalculated to account for the nitrogen dilution effect:

(19) $$P_{O_{2}}=\frac{P_{C}-R_{HC}P_{H_{2}O}}{\left(1+\frac{0.79}{0.21}\right)\exp\;\left(\frac{0.291}{T^{0.832}}\frac{I}{A}\right)}$$

In the above expressions, $R_{HA}$ and $R_{HC}$ denote the relative humidity at the anode and cathode sides, $P_{A}$ and $P_{C}$ are the respective inlet pressures, *A* is the active cell area, *I* is the stack current, and $P_{H_{2}O}$ is the water vapor saturation pressure. The latter is computed using the following empirical polynomial, where ΔT=T−273.15:

(20) $$\begin{array}{cc} \log_{10}\left(P_{H_{2}O}\right) & =29.5\times 10^{-3}\Delta T-91.8\times 10^{-6}{\left(\Delta T\right)}^{2}\\ \; & \;+14.4\times 10^{-8}{\left(\Delta T\right)}^{3}-2.18 \end{array}$$

Electrode kinetics introduce an activation overpotential, $V_{act}$, resulting from sluggish charge-transfer reactions at both the anode and cathode catalyst layers. This loss is modeled through the following semi-empirical expression derived from Butler-Volmer kinetics:

(21) $$V_{act}=-\left[\xi_{1}+\xi_{2}T+\xi_{3}T\ln\left(C_{O_{2}}\right)+\xi_{4}T\ln\left(I\right)\right]$$

where $\xi_{1}$ through $\xi_{4}$ are empirical fitting parameters with foundations in thermodynamic and electrochemical theory, and $C_{O_{2}}$ is the dissolved oxygen concentration at the cathode catalyst interface. Applying Henry’s law, $C_{O_{2}}$ is related to the oxygen partial pressure by:

(22) $$C_{O_{2}}=\frac{P_{O_{2}}}{5.08\times 10^{6}}\exp\;\left(\frac{498}{T}\right)$$

Resistive losses within the cell manifest as the ohmic drop $V_{ohm}$, which arises from both membrane ionic resistance and interfacial contact resistance during proton transport:

(23) $$V_{ohm}=I\left(R_{m}+R_{c}\right)$$

Given the narrow operating temperature window of PEMFCs, the contact resistance $R_{c}$ is assumed constant. The membrane resistance $R_{m}$ is computed from its thickness *l*, active area *A*, and the membrane-specific resistivity $\rho_{m}$:

(24) $$R_{m}=\frac{\rho_{m}l}{A}$$

The resistivity $\rho_{m}$ accounts for the effects of current density and temperature on proton conductivity through the hydrated membrane:

(25) $$\rho_{m}=\frac{181.6\left(1+0.03\frac{I}{A}+0.062{\left(\frac{T}{303}\right)}^{2}{\left(\frac{I}{A}\right)}^{2.5}\right)}{\left(\lambda -0.634-3\frac{I}{A}\right)\exp\;\left(4.18\frac{T-303}{T}\right)}$$

here, λ is a membrane hydration parameter whose value is not known a priori and must be estimated through optimization.

Let the current density be defined as J=I/A. At high current densities, mass transport limitations reduce the availability of reactants at the reaction sites, giving rise to a concentration overpotential $V_{con}$:

(26) $$V_{con}=-\beta \ln\;\left(1-\frac{J}{J_{max}}\right)$$

where $J_{max}$ represents the limiting current density set by the reactant supply rate, and β is a cell-specific empirical coefficient reflecting operating conditions.

The complete voltage model involves seven parameters that cannot be determined analytically:

$$X=[\xi_{1},\;\xi_{2},\;\xi_{3},\;\xi_{4},\;\lambda ,\;\beta ,\;R_{c}]$$

These quantities are identified by fitting the model predictions to experimental polarization curves using metaheuristic optimization algorithms.

### 5.2. Objective Function Formulation

Within the electrochemical model formulated in Equations (14)–(26), two distinct categories of parameters can be identified. The first category encompasses the measurable operating variables; namely *T*, $R_{HA}$, $R_{HC}$, $P_{A}$, and $P_{C}$, these values are recorded directly during experimental testing and vary with the operating conditions. The partial pressures $P_{H_{2}}$ and $P_{O_{2}}$ are subsequently computed from these operating variables using the relations defined in Equations (17)–(19).

The second category consists of the seven unknown model parameters $\xi_{1},\xi_{2},\xi_{3},\xi_{4},\lambda ,\beta$, and $R_{c}$, which carry physical significance but cannot be measured directly and must instead be estimated from the experimental voltage-current (V–I) characteristic of the stack.

The identification of these unknowns is formulated as a minimization problem, where the goal is to find the parameter vector $x=[\xi_{1},\xi_{2},\xi_{3},\xi_{4},\lambda ,\beta ,R_{c}]$ that brings the model output into closest agreement with the measured polarization data. The quality of this agreement is quantified through a cost function built on the sum of squared errors (SSE) between experimental observations and model predictions across all available datasets. For a total of *N* measurement points, the fitness criterion to be minimized is defined as:

(27) $$\mathrm{SSE}\left(x\right)=\sum_{i=1}^{N}\varepsilon^{2}\left(i\right),\;\varepsilon \left(i\right)=V_{m}\left(i\right)-V_{c}\left(i,x\right)$$

where $V_{m}\left(i\right)$ denotes the experimentally recorded stack voltage, and $V_{c}\left(i,x\right)$ represents the voltage predicted by the mathematical model for a given parameter set *x*. The optimal solution is the vector $x^{*}$ that globally minimizes SSE(x), a task delegated to metaheuristic optimization algorithms owing to the nonlinear and multimodal nature of the search space.

It should be noted that this study focuses on static parameter identification from a single polarization curve under fixed operating conditions. Because PEMFC internal parameters vary over time as the constituent materials degrade, online or adaptive parameter tracking using sequential polarization data is required to monitor these time-varying characteristics and is beyond the scope of the present study.

## 6. Results and Discussion of PEMFC Parameter Identification

The selected stacks considered in this study are the NedStack PS6 and the Ballard Mark V PEMFC stacks. To enable a meaningful comparison, the main specifications of these two stacks are summarized as follows.

The NedStack PS6 stack consists of 65 cells with an active cell area of 240 cm^2^ and a membrane thickness of 178 µm. It operates at a temperature of 343 K and reaches a maximum current density of 5 A/cm^2^, with an operating partial pressure $P_{H_{2}O}$ and $P_{O_{2}}$ equal to 1. Similarly, the Ballard Mark V stack is composed of 35 cells with an active area of 50.6 cm^2^ per cell and the same membrane thickness of 178 µm. This stack also operates at 343 K with a partial pressure $P_{H_{2}O}$ and $P_{O_{2}}$ equal to 1, while its maximum current density reaches 1.50 A/cm^2^. These specifications provide the necessary baseline parameters used for the modeling and comparative analysis in this work [6,23].

The lower and upper bounds associated with the optimization problem are presented in Table 9.

For a fair comparison between the EOOA and OOA algorithms, the population size was set to 50 and the maximum number of iterations was fixed at 1000. Both algorithms were executed for 30 independent runs using MATLAB R2021a on a personal computer equipped with an Intel Core i5-3230M CPU operating at 2.60 GHz and 8 GB of RAM.

**Figure 7:**
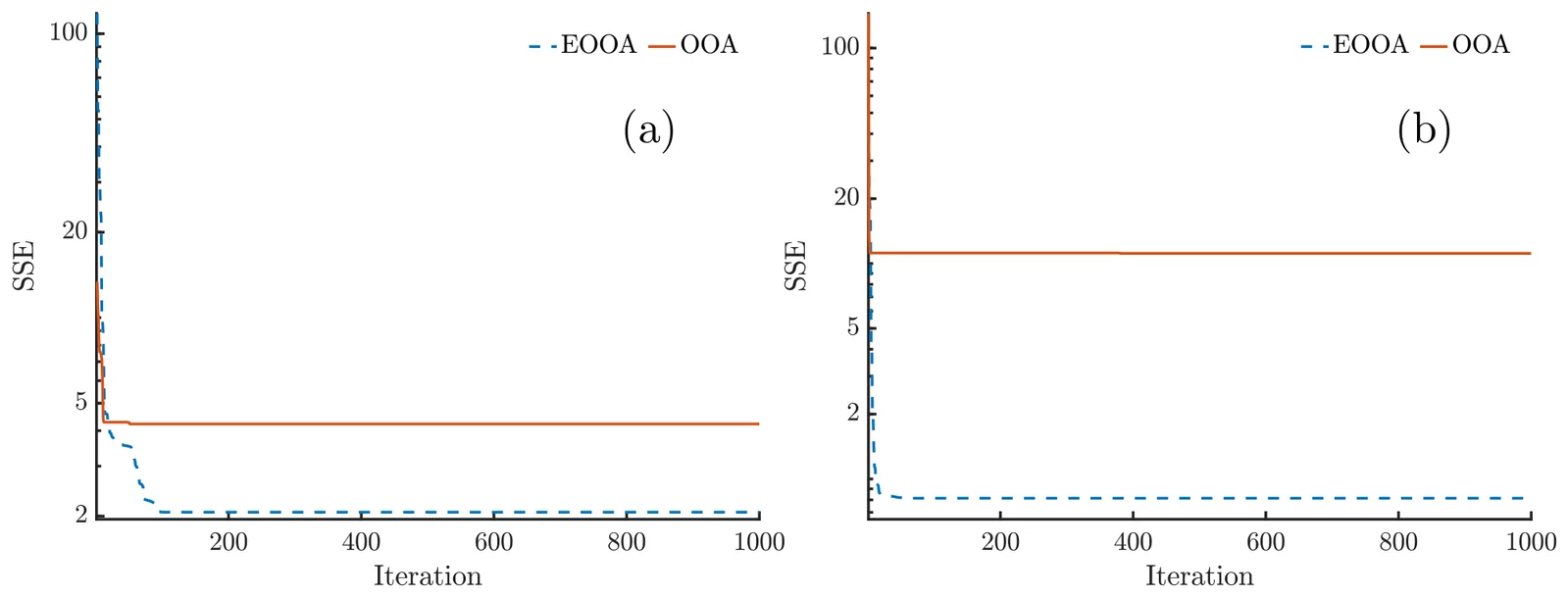
The convergence curves of EOOA and OOA for the parameter extraction process of (**a**) NedStack PS6 and (**b**) Ballard Mark V PEMFC stacks.

The convergence curves of EOOA and OOA are illustrated in Figure 7 for both PEMFC stacks.

The proposed EOOA demonstrates a significantly superior convergence behavior compared to the original OOA across both PEMFC stacks. For the NedStack PS6 stack (Figure 7a), EOOA achieves a rapid and steep reduction in the SSE within the first 100 iterations, ultimately converging to a much lower SSE value of approximately 2, whereas OOA stagnates at a considerably higher SSE of around 4, indicating that it becomes trapped in a local optimum. A similar behavior is observed for the Ballard Mark V PEMFC stack (Figure 7b), where EOOA converges swiftly to a near-optimal SSE close to 1, while OOA fails to escape its initial solution and remains at a high SSE of approximately 11 throughout the entire optimization process. These results clearly demonstrate the enhanced global search capability and exploitation efficiency of the proposed EOOA, confirming its superiority in extracting accurate parameters for PEMFC models compared to the standard OOA.

Table 10 presents the statistical comparison of the final SSE values obtained by the proposed EOOA and the original OOA for the parameter extraction of the NedStack PS6 and Ballard Mark V PEMFC stacks.

Theses results confirm the superior stability and accuracy of the proposed EOOA compared to OOA for both PEMFC stacks. For the NedStack PS6 stack, EOOA achieves identical best, worst, mean, and median SSE values of 2.06555691, with an extremely small standard deviation of $4.249\times 10^{-15}$. This behavior demonstrates that EOOA consistently converges to the same optimal solution regardless of the initial population. In contrast, OOA shows a large variation in performance, with SSE values ranging from 4.22307487 to 143.721555 and a high standard deviation of 39.16343, indicating unstable convergence behavior.

Similarly, for the Ballard Mark V stack, EOOA maintains identical statistical indicators with best, worst, mean, and median SSE values of 0.813911703, and a negligible standard deviation ($1.17295832\times 10^{-15}$), confirming its strong robustness and reliability. On the other hand, OOA exhibits significant variability, with SSE values ranging from 11.1423984 to 282.501137, a mean value of 96.272388, and a large standard deviation of 57.9321116. These results highlight that EOOA not only achieves better accuracy but also provides highly consistent solutions across multiple runs, demonstrating its effectiveness and robustness for PEMFC parameter extraction compared to the original OOA.

**Figure 8:**
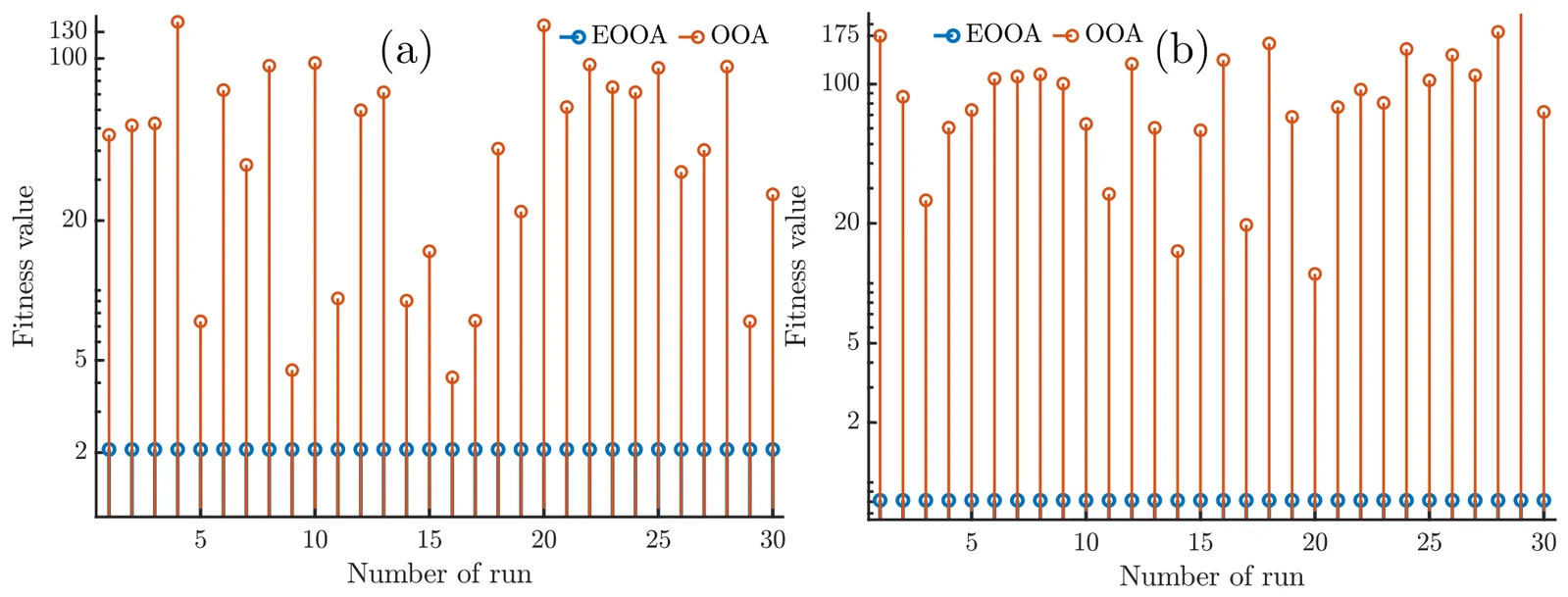
Fitness values obtained from each run of the EOOA and OOA for: (**a**) NedStack PS6 and (**b**) Ballard Mark V PEMFC stacks. (Note: the hiding value of OOA at run 29 of (**b**) is about 282.5).

Figure 8 report the best fitness values obtained so far for EOOA and OOA for both investigated PEMFC stacks for the 30 independent runs. EOOA achieves identical fitness values across all 30 runs for both the NedStack PS6 and Ballard Mark V stacks, indicating excellent stability and consistent convergence to the optimal solution. In contrast, OOA exhibits large variations between runs, with widely scattered fitness values and occasional extremely high SSE values. These results confirm that EOOA provides more reliable and robust performance than OOA for PEMFC parameter extraction.

The optimal parameter values obtained using EOOA and OOA are summarized in Table 11, alongside with the results reported in the literature using several recent metaheuristic algorithms including, flood algorithm (FLA) [6], cloud drift optimization (CDO) algorithm [22], artificial rabbits optimization algorithm (ARO) [25], and circle search algorithm (CSA) [24].

The results show that EOOA achieves the lowest SSE values for both PEMFC stacks, indicating a highly accurate parameter estimation. For the NedStack PS6 stack, EOOA attains an SSE of 2.06555691, matching the best results reported in the literature while outperforming OOA. Similarly, for the Ballard Mark V stack, EOOA reaches an SSE of 0.813911703, which is equal to or better than most reported methods. In contrast, the original OOA produces significantly larger SSE values for both stacks, confirming the effectiveness of the proposed enhancements in improving optimization accuracy.

**Figure 9:**
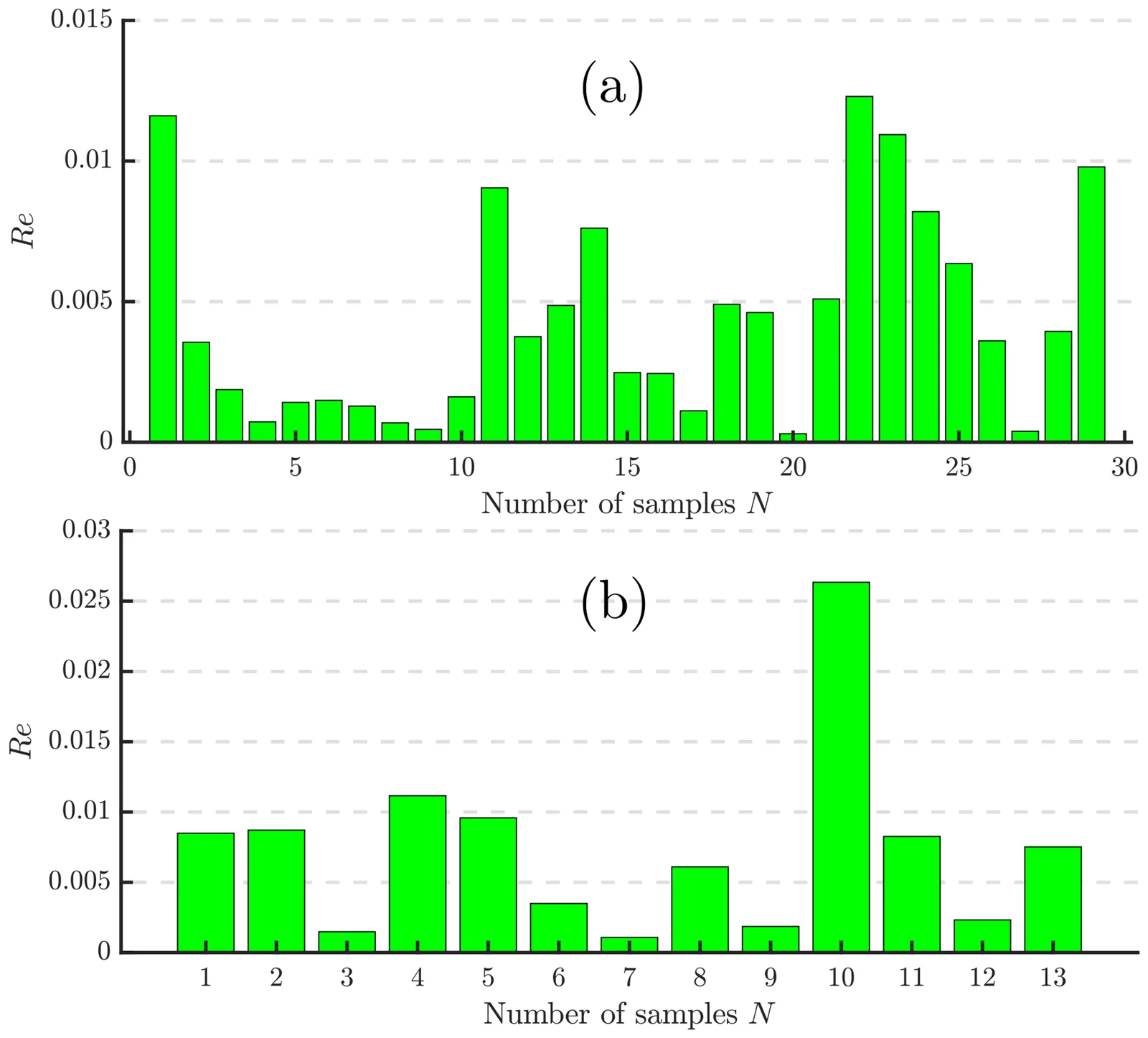
Relative error based on the parameters obtained by EOOA for: (**a**) NedStack PS6 and (**b**) Ballard Mark V PEMFC stacks.

Figure 9 presents the relative error defined by (28) for the NedStack PS6 and Ballard Mark V PEMFC stacks using the parameters extracted by EOOA.

(28) $$Re=\frac{V_{m}-V_{c}}{V_{m}}$$

The relative error Re measures the deviation between the experimental voltage and the voltage predicted by the PEMFC model. As shown in Figure 9, the errors for the NedStack PS6 stack remain very small, mostly below 0.010, indicating high model accuracy. Similarly, the Ballard Mark V stack exhibits low relative errors across most samples, with a maximum value of about 0.026, the small error values confirm that the parameters obtained by EOOA accurately reproduce the experimental data for both PEMFC stacks.

**Figure 10:**
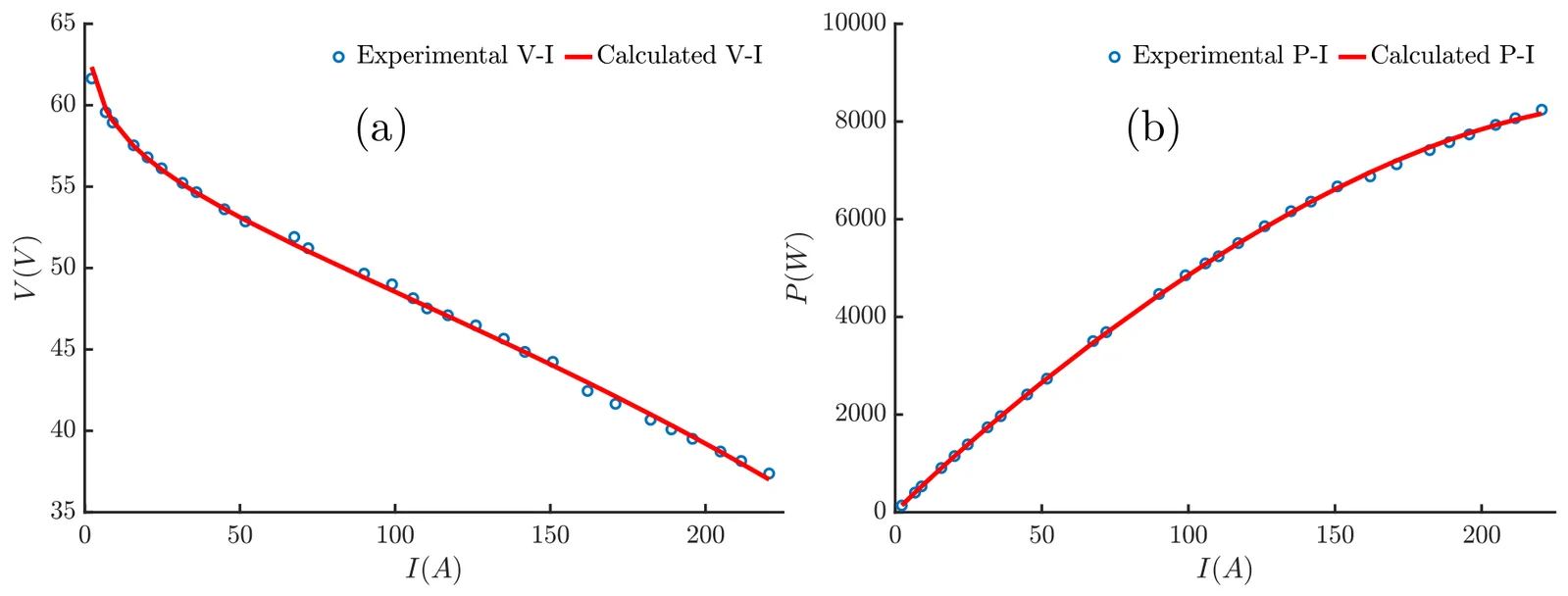
Experimental vs. Calculated (**a**) I–V and (**b**) P–V polarization curves using EOOA for NedStack PS6 PEMFC stack.

**Figure 11:**
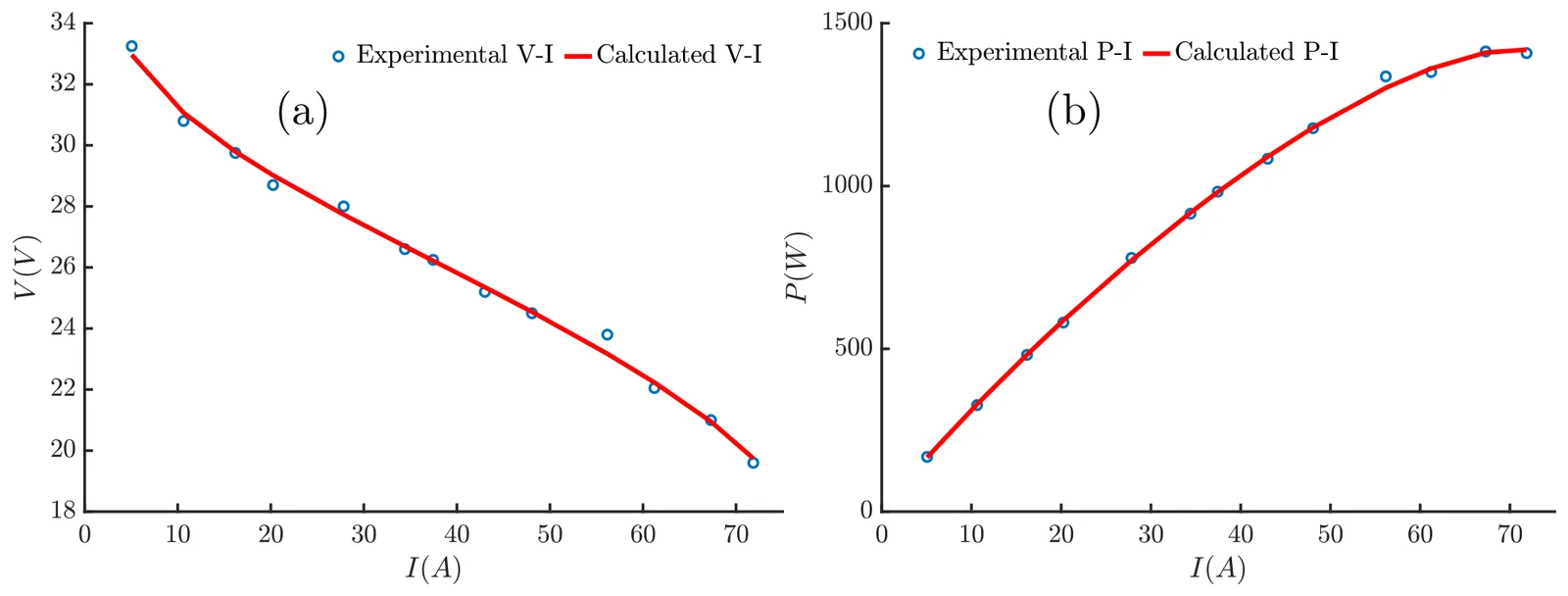
Experimental vs. Calculated (**a**) I–V and (**b**) P–V polarization curves using EOOA for Ballard Mark V PEMFC stack.

Based on the parameters extracted using EOOA, the estimated and experimental V–I and P–I polarization curves are compared in Figure 10 and Figure 11.

The calculated curves closely match the experimental data for the NedStack PS6 stack across the entire current range, accurately capturing the nonlinear voltage drop and corresponding power output. A similar agreement is observed for the Ballard Mark V stack in Figure 11, where the model predictions closely follow the measured V–I and P–I characteristics. This strong correspondence confirms that the parameters identified by EOOA enable an accurate representation of the PEMFC polarization behavior.

**Figure 12:**
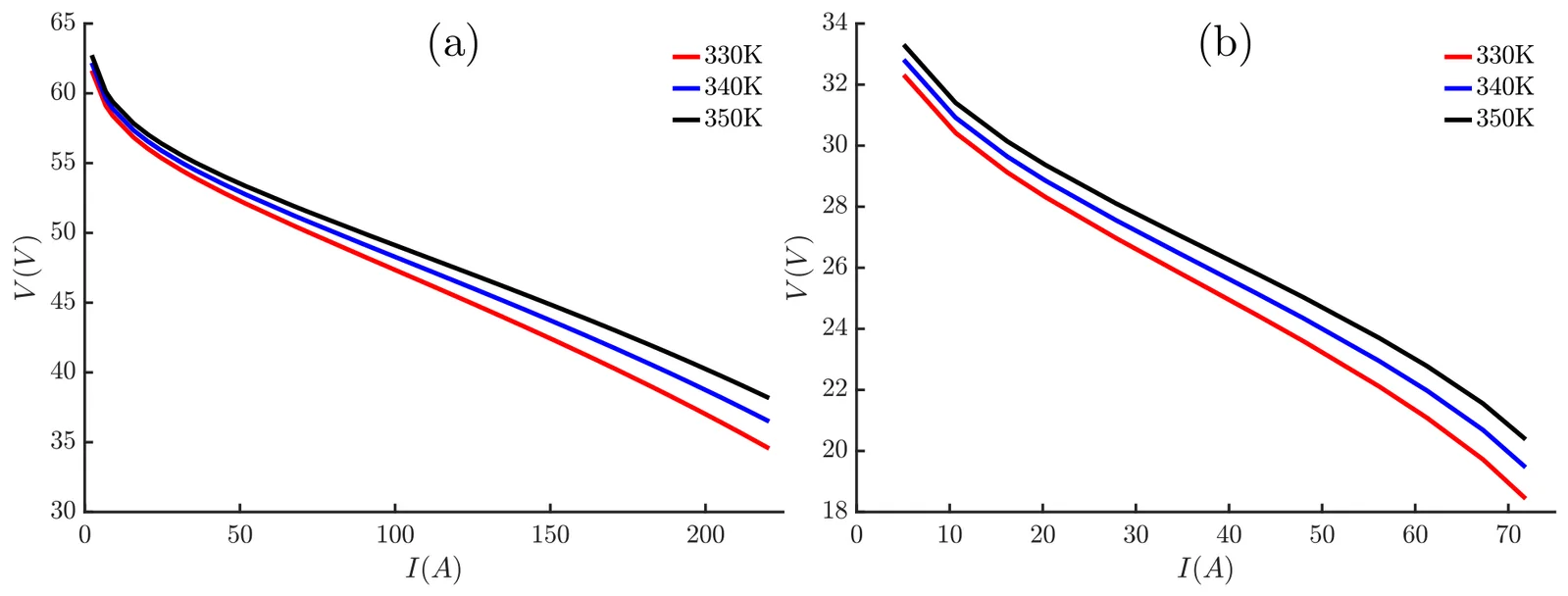
I–V polarization curves using EOOA for (**a**) NedStack PS6 and (**b**) Ballard Mark V PEMFC stacks under variable temperature.

**Figure 13:**
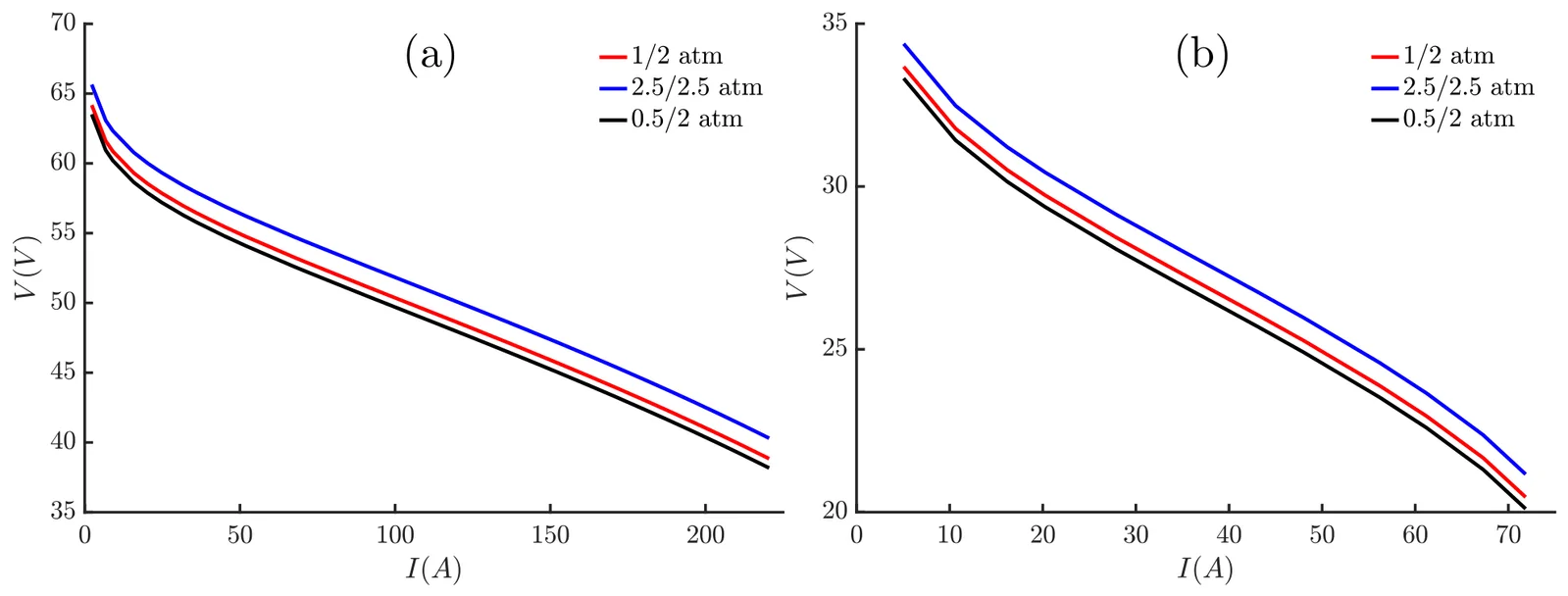
I–V polarization curves using EOOA for (**a**) NedStack PS6 and (**b**) Ballard Mark V PEMFC stacks under variable pressure.

Figure 12 and Figure 13 illustrate the V–I polarization curves of the NedStack PS6 and Ballard Mark V PEMFC stacks under different temperature and pressure conditions using the parameters identified by EOOA.

Figure 12 shows the polarization curves at temperatures of 330 K, 340 K, and 350 K. For both stacks, the voltage increases slightly with temperature over the entire current range. This improvement is due to enhanced electrochemical reaction kinetics and higher membrane proton conductivity at elevated temperatures, which reduce internal losses and improve stack performance.

Figure 13 presents the polarization curves under different anode/cathode pressure conditions. Higher reactant pressures result in higher stack voltages, with the 2.5/2.5 atm condition providing the best performance. This is attributed to increased reactant partial pressures, which enhance the Nernst potential and reduce concentration losses.

The results demonstrate that the EOOA-based model accurately reflects the expected effects of temperature and pressure variations on PEMFC performance.

## 7. Conclusions

This paper examined two main aspects. The first aspect was the proposal of a new algorithm, an enhanced version of the Osprey Optimization Algorithm (OOA), called the Enhanced Osprey Optimization Algorithm (EOOA). The second aspect involved applying the EOOA to extract the parameters of two commercial PEMFC stacks, namely NedStack PS6 and Ballard Mark V.

The results of this study confirm that the EOOA outperforms the conventional OOA and four other optimization algorithms. These algorithms were evaluated using the CEC2022 benchmark functions, where the EOOA ranked first in all functions except $F_{10}$, in which it ranked third. Furthermore, statistical analysis indicates that the EOOA significantly outperforms the compared algorithms.

The parameter extraction analysis for PEMFC stacks revealed that the EOOA achieved a global minimum sum of squared errors (SSE) of approximately 2 for the NedStack PS6 and about 1 for the Ballard Mark V. These results demonstrate that the proposed algorithm provides better performance compared to the OOA and other algorithms reported in the literature for the same problem.

This work highlights the importance of continued research on the development of novel optimization algorithms and the enhancement of existing ones to address challenging optimization problems more effectively. Beyond PEMFC electrical parameter identification, the general-purpose optimization framework of the proposed EOOA makes it suitable for a wide range of fuel-cell optimization tasks, including optimizing catalyst-layer composition and loading using surrogate activity models, tuning machine-learning models for catalyst performance prediction based on structural descriptors, and estimating kinetic parameters of catalyst-layer models from experimental cyclic voltammetry data. Future research should investigate the applicability of EOOA to a broader range of PEMFC stacks and other engineering optimization problems. In addition, integrating advanced population initialization strategies and adaptive parameter-control mechanisms may further enhance its convergence behavior, robustness, and overall optimization performance.

## Figures and Tables

**Table 1:** Summary of OOA modifications and applications.

Ref.	Year	Main Modifications to OOA	Applications
[44]	2023	Sobol sequence initialization + Weibull step factor + Firefly-inspired disturbance	Reactive power optimization (IEEE 33 & 69-bus networks)
[36]	2024	Lévy flight	Task scheduling in cloud computing
[45]	2024	Lévy flight + Brownian motion + RFDB selection	CEC2017, CEC2022, and engineering design optimization
[46]	2024	Circle chaotic mapping + elite guidance mechanism + dynamic chaotic weight	21 benchmark test functions, CEC2022, the LSTM power load forecasting problem, and engineering design optimization.
[47]	2024	Logistic chaotic map + good point set + two-color group strategies + Harris Hawks + the strolling and wandering strategy + the optimized spiral search and firefly perturbation	classical benchmark functions, CEC2020, CEC2022, and engineering design optimization
[48]	2024	Fuch chaotic mapping + adaptive weighting + Cauchy mutation + Sparrow warner mechanism	Three-bar truss design, CEC2017, and classical benchmarks
[49]	2024	Piecewise chaotic map + adaptive Lévy exploration + trigonometric disturbance + nonlinear control parameters	UAV path planning
[38]	2024	Attack-defense strategy (ADSOOA)	PEMFC electrochemical parameter identification
[43]	2024	Modified OOA for CapsNet hyperparameter tuning	Leukemia image classification
[41]	2024	Hybrid with Kookaburra Optimization Algorithm (KOU)	Adaptive load balancing in distributed cloud
[42]	2024	Promoted Osprey Optimizer (POO): diving, soaring & gliding strategies	Optimal reactive power dispatch with EV integration
[39]	2025	Tent mapping initialization + Gaussian distribution position update	Mobile robot path planning
[37]	2025	Lévy flight	Fuzzy availability optimization of steel manufacturing unit
[40]	2025	Hybrid with Parrot Optimizer (OOA + PO)	Supercapacitor parameter identification
[50]	2025	Quasi-oppositional learning + DE mutation + Brownian motion	Off-grid hybrid renewable energy system sizing

**Table 2:** CEC 2022 benchmark functions. $F_{min}$ is the global minimum of the problem. Bias is the bias value.

No.	Name	$F_{\min}$	Bias
$F_{1}$	Shifted and full rotated Zakharov function	0	300
$F_{2}$	Shifted and rotated Rosenbrock’s function	0	400
$F_{3}$	Shifted and fully rotated Rastrigin’s function	0	600
$F_{4}$	Shifted and fully rotated non-continuous Rastrigin’s function	0	800
$F_{5}$	Shifted and fully rotated Levy function	0	900
$F_{6}$	Hybrid function 1 (n=3)	0	1800
$F_{7}$	Hybrid function 2 (n=6)	0	2000
$F_{8}$	Hybrid function 3 (n=5)	0	2200
$F_{9}$	Composition function 1 (n=5)	0	2300
$F_{10}$	Composition function 2 (n=4)	0	2400
$F_{11}$	Composition function 3 (n=5)	0	2600
$F_{12}$	Composition function 4 (n=6)	0	2700
	Bounds: $\left[-100,100\right]$, Dimension: 10		

**Table 3:** Control Parameters of the Optimization Algorithms.

Algorithm	Parameter	Value
EOOA	λ	3
	CR	0.9
GA	Distribution index for crossover	20
	Distribution index for mutation	20
	Probability of crossover	0.8
	Probability of mutation	0.2
SCA	Convergence parameter (*a*)	Linear reduction [2→0]
ABC	Percentage of onlooker bees	50% of the colony
	Number of scout bees	1
HHO	Initial energy ($E_{0}$)	$E_{0}\in \left[-1,1\right]$

**Table 4:** Statistical results of the EOOA and other comparison algorithms on CEC2022.

	Metric	EOOA	OOA	GA	SCA	ABC	HHO
$F_{1}$	Best	300.00	4153.80	7130.98	907.40	4054.04	304.15
	Worst	312.63	15,007.39	38,997.24	4319.24	13,844.97	1006.83
	Mean	**300.43**	8733.37	22,824.76	2023.08	8711.75	432.05
	Std	2.30	3018.39	7400.76	757.44	2024.01	171.31
	Rank	1	5	6	3	4	2
$F_{2}$	Best	400.00	601.43	825.22	431.33	406.52	400.10
	Worst	472.28	1849.70	5158.27	500.72	425.55	585.28
	Mean	**411.63**	1161.99	2033.73	471.08	415.03	441.29
	Std	20.60	340.69	1094.35	17.36	5.32	49.16
	Rank	1	5	6	4	2	3
$F_{3}$	Best	600.01	620.84	629.19	615.09	601.33	604.52
	Worst	606.03	652.87	669.82	632.51	604.82	661.47
	Mean	**601.46**	634.90	648.96	622.49	602.75	636.57
	Std	1.69	7.60	10.59	4.10	0.92	13.07
	Rank	1	4	6	3	2	5
$F_{4}$	Best	802.98	820.54	835.85	833.84	823.16	813.13
	Worst	828.85	855.33	893.07	855.69	844.99	843.89
	Mean	**817.15**	835.82	859.10	844.18	832.46	826.59
	Std	6.45	8.38	12.98	6.08	5.32	8.09
	Rank	1	4	6	5	3	2
$F_{5}$	Best	900.45	1017.33	1210.92	946.66	920.79	1095.33
	Worst	989.73	1546.90	3359.77	1267.38	1762.41	1545.87
	Mean	**921.03**	1208.70	2027.79	1032.06	1152.37	1341.39
	Std	27.27	136.68	481.94	72.65	178.89	114.20
	Rank	1	4	6	2	3	5
$F_{6}$	Best	1802.17	1868.47	1,456,569.57	323,388.89	3594.06	1933.80
	Worst	1882.21	9,652,692.22	146.31 × 10^7^	8,267,935.34	262,482.82	14,600.85
	Mean	**1837.13**	682,556.85	131.37 × 10^6^	2,869,825.05	31,194.54	4457.14
	Std	21.77	2,140,492.33	284.66 × 10^6^	1,884,078.39	47,409.99	2849.03
	Rank	1	4	6	5	3	2
$F_{7}$	Best	2000.00	2039.24	2032.50	2047.63	2013.89	2028.16
	Worst	2043.39	2112.92	2176.37	2079.77	2031.46	2140.29
	Mean	**2020.94**	2072.40	2101.47	2063.07	2024.94	2068.44
	Std	8.13	18.12	34.13	8.07	4.40	32.03
	Rank	1	5	6	3	2	4
$F_{8}$	Best	2200.24	2223.02	2229.91	2222.75	2214.80	2211.15
	Worst	2222.78	2252.09	2410.68	2238.21	2224.18	2264.86
	Mean	**2213.95**	2230.01	2277.15	2233.61	2220.75	2230.60
	Std	8.90	5.24	54.21	3.30	3.24	11.44
	Rank	1	3	6	5	2	4
$F_{9}$	Best	2529.28	2644.02	2568.60	2541.63	2445.25	2534.55
	Worst	2529.28	2780.78	3105.80	2605.56	2554.98	2664.75
	Mean	**2529.28**	2715.25	2772.33	2572.82	2532.68	2586.29
	Std	2.53 × 10^−13^	33.39	100.57	18.13	18.55	32.33
	Rank	1	5	6	3	2	4
$F_{10}$	Best	2500.26	2508.10	2517.91	2501.20	2437.25	2438.71
	Worst	2632.76	3467.20	3716.79	2652.86	2501.28	2727.78
	Mean	2528.62	2657.11	2730.19	2507.40	**2494.22**	2578.91
	Std	51.92	170.14	286.15	27.49	14.88	76.83
	Rank	3	5	6	2	1	4
$F_{11}$	Best	2600.00	2895.83	2869.67	2757.64	2726.08	2604.40
	Worst	2912.72	4285.00	5104.47	2841.59	2760.86	3212.27
	Mean	**2645.52**	3437.13	3885.61	2779.83	2749.42	2786.61
	Std	90.78	373.06	611.33	14.86	9.09	173.43
	Rank	1	5	6	3	2	4
$F_{12}$	Best	2859.37	2939.17	2897.90	2867.15	2865.45	2867.82
	Worst	2869.38	3179.70	3205.87	2873.59	2871.24	2998.13
	Mean	**2865.48**	3028.71	3020.57	2870.73	2868.08	2906.15
	Std	2.16	64.21	79.34	1.53	1.43	38.41
	Rank	1	6	5	3	2	4
Mean rank		1.16	4.58	5.92	3.42	2.33	3.58
Final rank		1	5	6	3	2	4

**Table 5:** The Wilcoxon rank sum test *p*-values between the EOOA and other algorithms on CEC2022.

	OOA		GA		SCA		ABC		HHO	
$F_{1}$	3.02 × 10^−11^	+	3.02 × 10^−11^	+	3.02 × 10^−11^	+	3.02 × 10^−11^	+	6.07 × 10^−11^	+
$F_{2}$	2.91 × 10^−11^	+	2.91 × 10^−11^	+	3.72 × 10^−9^	+	1.70 × 10^−6^	+	0.000493071	+
$F_{3}$	3.02 × 10^−11^	+	3.02 × 10^−11^	+	3.02 × 10^−11^	+	0.000168132	+	3.69 × 10^−11^	+
$F_{4}$	3.81 × 10^−10^	+	3.01 × 10^−11^	+	3.01 × 10^−11^	+	2.15 × 10^−10^	+	1.99 × 10^−5^	+
$F_{5}$	3.02 × 10^−11^	+	3.02 × 10^−11^	+	3.82 × 10^−10^	+	5.07 × 10^−10^	+	3.02 × 10^−11^	+
$F_{6}$	4.50 × 10^−11^	+	3.02 × 10^−11^	+	3.02 × 10^−11^	+	3.02 × 10^−11^	+	3.02 × 10^−11^	+
$F_{7}$	3.34 × 10^−11^	+	4.08 × 10^−11^	+	3.02 × 10^−11^	+	0.005322078	+	1.61 × 10^−10^	+
$F_{8}$	3.02 × 10^−11^	+	3.02 × 10^−11^	+	3.34 × 10^−11^	+	0.000587373	+	1.17 × 10^−9^	+
$F_{9}$	3.16 × 10^−12^	+	3.16 × 10^−12^	+	3.16 × 10^−12^	+	1.54 × 10^−9^	+	3.16 × 10^−12^	+
$F_{10}$	3.65 × 10^−8^	+	6.53 × 10^−7^	+	0.00033679	−	0.795845542	=	5.27 × 10^−5^	+
$F_{11}$	4.08 × 10^−11^	+	3.69 × 10^−11^	+	8.48 × 10^−9^	+	1.61 × 10^−6^	+	1.47 × 10^−7^	+
$F_{12}$	3.01 × 10^−11^	+	3.01 × 10^−11^	+	1.46 × 10^−10^	+	3.32 × 10^−6^	+	4.49 × 10^−11^	+

**Table 6:** Mean CPU execution time (in seconds, averaged over 30 independent runs) for EOOA and the compared algorithms on the CEC2022 benchmark functions.

Function	EOOA	OOA	GA	SCA	ABC	HHO
$F_{1}$	0.7490	0.3664	0.2333	0.1901	0.5159	0.4200
$F_{2}$	0.6510	0.3359	0.2142	0.1720	0.5182	0.3994
$F_{3}$	0.9620	0.5091	0.3139	0.2590	0.7393	0.6526
$F_{4}$	0.8454	0.4441	0.2994	0.2345	0.6729	0.5488
$F_{5}$	0.7056	0.3703	0.2324	0.1982	0.5594	0.4724
$F_{6}$	0.6637	0.3312	0.2155	0.1738	0.5146	0.4270
$F_{7}$	0.8997	0.4883	0.2955	0.2540	0.6695	0.6184
$F_{8}$	0.9795	0.5434	0.3246	0.2851	0.7369	0.6904
$F_{9}$	0.8368	0.4588	0.2824	0.2415	0.6553	0.5567
$F_{10}$	0.8070	0.4327	0.2680	0.2311	0.6388	0.5518
$F_{11}$	0.9456	0.5251	0.3125	0.2726	0.7255	0.6509
$F_{12}$	0.9614	0.5391	0.3200	0.2829	0.7495	0.6663

**Table 7:** Sensitivity analysis of EOOA with respect to λ and Cr on $F_{4}$, $F_{6}$, and $F_{9}$ (CEC2022, Dim = 10).

Function	Metric	λ=2			λ=3			λ=4		
		Cr=0.1	Cr=0.5	Cr=0.9	Cr=0.1	Cr=0.5	Cr=0.9	Cr=0.1	Cr=0.5	Cr=0.9
$F_{4}$	Mean	817.837	815.820	816.782	815.688	815.753	816.151	816.491	818.407	816.251
	Std	4.888	6.633	6.463	6.053	5.890	4.886	5.286	6.862	5.630
	Rank	8	3	7	1	2	4	6	9	5
$F_{6}$	Mean	2794.839	2520.696	1840.618	2724.433	2532.162	1848.347	2769.823	3032.449	1860.289
	Std	1237.722	748.978	46.159	809.589	985.200	21.341	1181.660	1538.292	52.776
	Rank	8	4	1	6	5	2	7	9	3
$F_{9}$	Mean	2586.390	2529.284	2534.182	2585.902	2534.182	2529.284	2592.355	2529.284	2529.284
	Std	25.917	$2.30\times 10^{-11}$	26.826	29.615	26.826	$3.48\times 10^{-13}$	25.356	$3.07\times 10^{-6}$	$1.04\times 10^{-12}$
	Rank	8	3	5	7	6	1	9	4	2
Average Rank		8.00	3.33	4.33	4.67	4.33	2.33	7.33	7.33	3.33
Overall Rank		9	2	4	6	5	1	7	8	3

**Table 8:** Mean CPU execution time (in seconds, averaged over 30 independent runs) for EOOA and OOA under the equal-FES.

Function	EOOA (s)	OOA (s)	Ratio (EOOA/OOA)
$F_{4}$	0.2264	0.1622	1.40
$F_{6}$	0.1805	0.1379	1.31
$F_{9}$	0.2300	0.1888	1.22

**Table 9:** Maximum and minimum bounds of the optimization problem.

	$\xi_{1}$	$\xi_{2}\times 10^{-3}$	$\xi_{3}\times 10^{-5}$	$\xi_{4}\times 10^{-3}$	λ	β(V)	$R_{c}\times 10^{-4}\;\left(\Omega \right)$
LB	−1.19969	1	3.6	−2.6	10	0.0136	1
UB	−0.8532	5	9.8	−0.954	24	0.5	8

**Table 10:** Statistical comparison of final SSE values for parameter extraction of the NedStack PS6 and Ballard Mark V PEMFC stacks using EOOA and OOA.

	NedStack PS6		Ballard Mark V	
	EOOA	OOA	EOOA	OOA
Best	2.06555691	4.22307487	0.813911703	11.1423984
Worst	2.06555691	143.721555	0.813911703	282.501137
Mean	2.06555691	52.4060547	0.813911703	96.272388
Median	2.06555691	49.1578444	0.813911703	90.1843350
std	$4.24916392\times 10^{-15}$	39.16343	$1.17295832\times 10^{-15}$	57.9321116

**Table 11:** Optimal parameter values for the NedStack PS6 and Ballard Mark V PEMFC stacks obtained using various algorithms.

Stack	Algorithm	$\xi_{1}$	$\xi_{2}\times 10^{-3}$	$\xi_{3}\times 10^{-5}$	$\xi_{4}\times 10^{-5}$	λ	$\beta \times 10^{-2}$ (V)	$R_{c}\times 10^{-4}$ (Ω)	SSE
NedStack PS6	EOOA	−0.8532054885	3.2473805394	9.6672550832	−9.54	12.5743308	1.36	1.00	2.06555691
	OOA	−0.8532	2.6985197306	5.7034876876	−9.5431452789	18.52727889	2.2673419287	2.5350701478	4.22307487
	FLA [6]	−1.19969	4.2686233306	9.7464882196	−9.54	12.5743308	1.36	1.00	2.06555691
	CDO [22]	−1.13625	3.22286	3.6	−9.54	13.323	1.36	1.00	2.10025
	ARO [25]	−1.008511	3.0434	4.9796	−9.54	13.445704	1.36	1.00	2.1112503
Ballard Mark V	EOOA	−1.015303253	3.4331835074	6.418427814	−16.725137846	24	1.5884374195	1.00	0.813911703
	OOA	−0.9968999021	3.7153844268	9.2894037787	−10.444184157	20.08769978	3.8813905461	3.3356796805	11.1423984
	FLA [6]	−1.199260732	4.4318028525	9.7231857179	−16.725137909	24	1.5884374024	1.00	0.813911703
	CSA [24]	−1.181342405	3.56096	3.9929	−16.2830	23	1.36	1.00	0.853607516
	ARO [25]	−1.158859	3.5208	4.0526	−16.7251	23.99	1.5884	1.00	0.8139117035

## Data Availability

The data presented in this study are available from the corresponding author upon reasonable request.
